# *In situ* Gelling Amphotericin B Nanofibers: A New Option for the Treatment of Keratomycosis

**DOI:** 10.3389/fbioe.2020.600384

**Published:** 2020-12-21

**Authors:** Benedikt Göttel, Henrike Lucas, Frank Syrowatka, Wolfgang Knolle, Judith Kuntsche, Joana Heinzelmann, Arne Viestenz, Karsten Mäder

**Affiliations:** ^1^Pharmaceutical Technology and Biopharmacy, Institute of Pharmacy, Martin-Luther University Halle-Wittenberg, Halle, Germany; ^2^Interdisciplinary Center of Materials Science, Martin-Luther University Halle-Wittenberg, Halle, Germany; ^3^Leibniz Institute of Surface Engineering (IOM), Leipzig, Germany; ^4^Department of Physics, Chemistry and Pharmacy, University of Southern Denmark, Odense, Denmark; ^5^Department of Ophthalmology, Martin Luther University Halle-Wittenberg, Halle, Germany

**Keywords:** keratomycosis, electrospinning, amphotericin B, polyelectrolyte complex, PLGA nanoparticle, *in situ* forming, hydrogel, ocular drug delivery

## Abstract

The purpose of our research was the development of Amphotericin B-loaded *in situ* gelling nanofibers for the treatment of keratomycosis. Different formulation strategies were applied to increase the drug load of the sparingly water-soluble Amphotericin B in electrospun Gellan Gum/Pullulan fibers. These include bile salt addition, encapsulation in poly(lactic-co-glycolic acid) (PLGA) nanoparticles and formation of a polymeric Amphotericin B polyelectrolyte complex. The Amphotericin B polyelectrolyte complex (AmpB-Eu L) performed best and was very effective against the fungal strain *Issatchenkia orientalis in vitro*. The complex was characterized in detail by attenuated total reflection infrared spectroscopy, X-ray powder diffraction, and differential scanning calorimetry. A heat induced stress test was carried out to ensure the stability of the polyelectrolyte complex. To gain information about the cellular tolerance of the developed polyelectrolyte complex a new, innovative multilayered-stratified human cornea cell model was used for determination of the cellular toxicity *in vitro*. For a safe therapy, the applied ophthalmic drug delivery system has to be sterile. Sterilization by electron irradiation caused not degradation of pure Amphotericin B and also for the bile salt complex. Furthermore, the developed Amphotericin B polyelectrolyte complex was not degraded by the irradiation process. In conclusion, a new polyelectrolyte Amphotericin B complex has been found which retains the antifungal activity of the drug with sufficient stability against irradiation-sterilization induced drug degradation. Furthermore, in comparison with the conventional used eye drop formulation, the new AmpB-complex loaded nanofibers were less toxic to cornea cells *in vitro*. Electrospinning of the Amphotericin B polyelectrolyte complex with Gellan Gum/ Pullulan leads to the formation of nanofibers with *in situ* gelling properties, which is a new and promising option for the treatment of keratomycosis.

## Introduction

Keratomycosis is a severe infection of the ocular surface and anterior segment of the eye (Bourcier et al., 2017). The disease is triggered by events such as wearing contact lenses, getting dirt into the eye or immunosuppressive drug therapies. Epidemiologic investigations show that 57% of the fungal keratitis patients wore contact lenses, 30% of these had an eye operation previously and 19% had jobs in the agriculture or gardener sections (Roth et al., 2019). The main challenging tasks in clinical practice are early and precise diagnoses, differentiation from other ocular infections, followed by an adequate therapy of the fungal strain which has to be performed in time, so that dramatic consequences like tissue damages and loss of sight can be prevented. In average, 32 days are necessary until the fungal infection of the eye is diagnosed (Roth et al., 2019). As consequence of the late diagnosis, keratomycosis is progressed and the therapy needs to be highly efficient for total patient recovery. An increased fungal keratitis incidence in countries with tropical climate paired with low-income regions in comparison with industrial countries is described in literature (Bharathi et al., 2007; Bhartiya et al., 2007; Green et al., 2008). Most keratitis infections are caused by *Fusarium* spp, *Candida* spp. and *Aspergillus* spp. (Nielsen et al., 2015). For fungal keratitis therapy, mainly Voriconazole and Amphotericin B (AmpB) are used solely or in combination via surface or anterior application therapy. The antimycotic therapy is characterized by high application frequency and high doses to reach complete recovery of the fungal infection (Behrens-Baumann et al., 2015; Farrell et al., 2017; Roth et al., 2019).

Because of its physicochemical properties, low solubility and stability in aqueous media, the biopharmaceutical classification system IV (BCS) class drug AmpB is still challenging to formulate for the treatment of fungal keratitis (Torrado et al., 2008). Until now, no licensed topical Amphotericin B formulation for the treatment of keratomycosis is available. Currently, off-label used drug delivery systems like eye drops and ointments are available. Eye drops are preferred, because of low costs, easy application, and high patient compliance. However, lid blink and rapid tear turnover limit the ocular residence time to only few minutes. Many systems contain bile salts as solubilizing excipients, which are characterized by side effects and cellular damages (Furrer et al., 2000, 2002). Lipophilic ointments increases the ocular residence time, but limit the oxygen supply of the cellular layers. As consequence, the tissue starts neovascularization (Holden et al., 1984; Stefansson et al., 1987).

**Figure 1:**
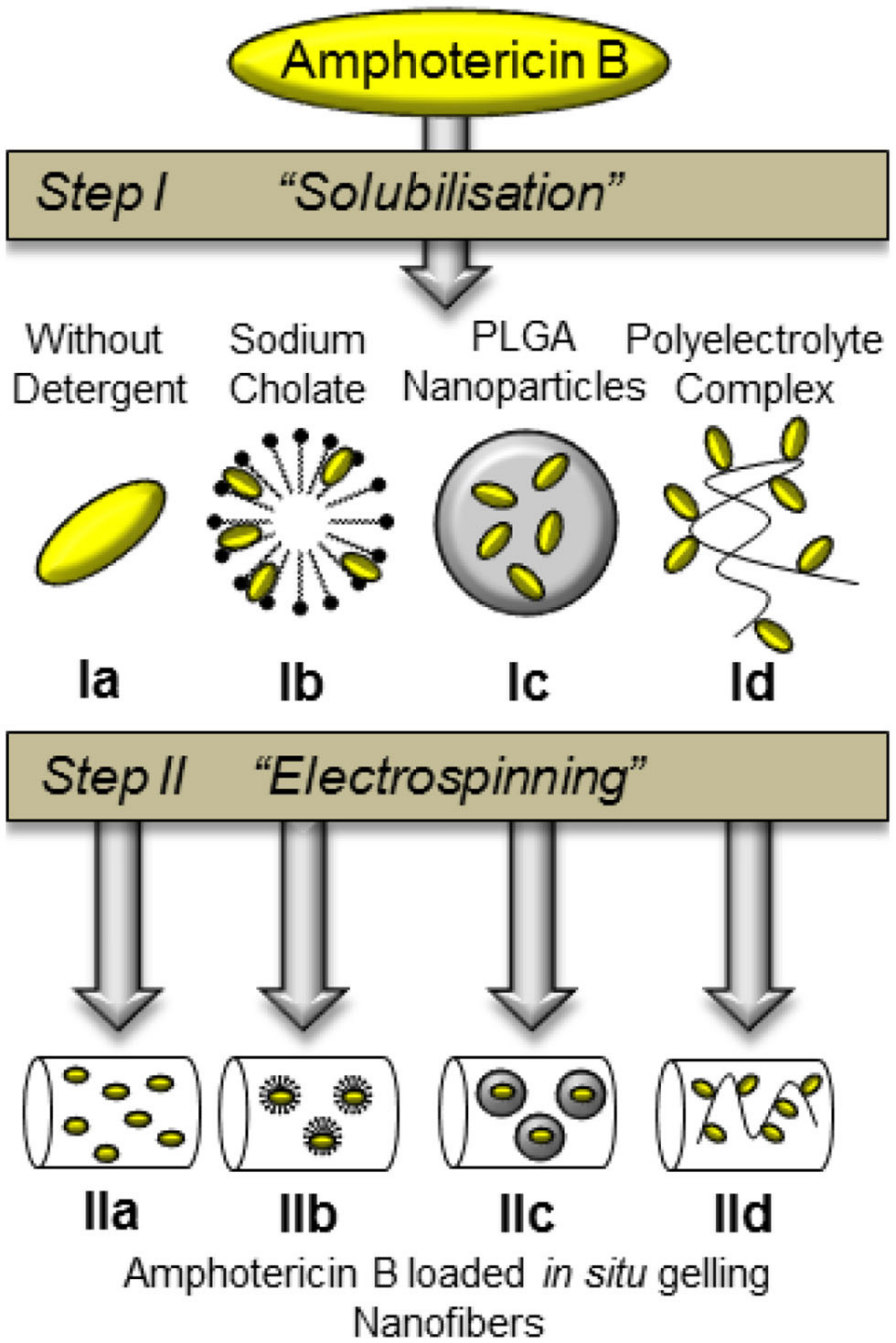
Schematic overview over different AmpB solubilisation techniques (Step I) and nanofiber compositions (Step II) with corresponding number of formulation.

Based on prior research, we developed solid *in situ* gelling nanofibers via electrospinning which were optimized for ocular administration. Nanofibers containing Gellan Gum LA as gelling agent and Pullulan as spinning copolymer were prepared by electrospinning (Göttel et al., 2020). The dry fibers were applied to the ocular surface and gel immediately after administration. Solid fibers have many advantages in comparison to conventional eye drops: The water free, solid state character increases stability and protects drugs from hydrolysis. Furthermore, the *in situ* gelling effect allows prolongation of the ocular residence time (Göttel et al., 2020). This formulation principle has the potential to decrease the administration frequency, the AmpB dose, and side effects by the lacrimal drainage. This aim of this study was to develop and to compare different AmpB formulations which ensure sufficient encapsulation of the drug into *in situ* gelling nanofibers. Therefore, the solubilisation of pure AmpB (Ia), AmpB with sodium cholate addition (Ib), AmpB-loaded nanoparticles (Ic), and a AmpB-polyelectrolyte complex (Id) were investigated in Step I. The solubilized drug was electrospun during Step II. A schematic overview over the solubilization techniques, preparation steps with the corresponding number of formulation are shown in Figure 1.

## Materials

Resomer RG 502 (PLGA) and Eudragit L (Eu-L) (Evonik Industries AG, Germany); Expansorb 5-88 PVA) (Merck KGaA, Germany); AmpB (Fagron GmbH & Co. KG, Germany); dimethylsulfoxide (DMSO) (Carl Roth GmbH + Co. KG, Germany), methanol (VWR Chemicals, Germany); sodium cholate (Sigma Aldrich, Germany); Pullulan was a gift form Nagase GmbH® (Germany); Kelcogel® GG-LA (CP-Kelco®, USA); sodium hydroxide (Grüssing GmbH, Germny); acetonitrile (VWR, Germany); disodium EDTA (Fluka Analytical, Switzerland); *Issatchenkia orientalis* (Leibnitz-Institute DSMZ German Collection of Microorganisms and Cell Cultures GmbH, Germany); potassium chloride (Grüssing GmbH, Germany); monopotassium phosphate (Grüssing GmbH, Germany); disodium phosphate (Grüssing GmbH, Germany); sodium bicarbonate (Grüssing GmbH, Germany); calcium chloride anhydrate (Grüssing GmbH, Germany); sodium chloride (Grüssing GmbH, Germany); sodium azide (Carl Roth, Germany).

Phosphate buffer pH 7.4 (PBS: 2.38 g Disodium phosphate, 0.19 g Monopotassium phosphate, 8 g Sodium chloride, 1,000 ml double distilled water). Simulated tear fluid pH 7.4 (STF: 6.8 g Sodium chloride, 1.4 g Potassium chloride, 2.2 g, Sodium bicarbonate, 0.06 g Calcium chloride anhydrate, 1,000 ml double distilled water). All buffer solutions were preserved with 0.02% sodium azide.

Universal medium for yeasts: 3 g Yeast extract, 3 g Malt extract (Carl Roth GmbH + Co. KG, Germany), 5 g Peptone form soybeans, 10 g Glucose^*^Monohydrate (Grüssing, Germany), 15 g Agar, 1,000 ml double distilled water (autoclaved).

## Methods

### Amphotericin B-Loaded PLGA Nanoparticles (Ic)

#### Preparation of PLGA Nanoparticles (Ic)

The preparation of PLGA nanoparticles (Ic) was performed by a modified method of Van de Ven et al. (2012). Thereby, PLGA was dissolved in DMSO, followed by addition of different amounts of AmpB until 30 mg/ml PLGA solutions with different drug-polymer mass ratios 1:100, 1:20, 1:10, and 2:10 were obtained. One milliliter drug-polymer solution was injected into 12.5 ml double distilled water stabilized with 0.5% poly(vinyl alcohol) (PVA). The polymer solution was injected with a flow rate of 50 μl/min and a 23 G needle into the aqueous media. The stabilized solution was stirred at 360 rpm with a magnetic stirrer. Five purification steps were performed by centrifugation to remove residual DMSO, stabilizer excess and non-entrapped AmpB. Five milliliters of the dispersion was diluted with 10 ml double distilled water, followed by transferring into a 100 kDa Centrifugal Filter (Amicon® Ultra 15 ml Centrifugal Filter, Merck Millipore) until the liquid level reached a minimal volume in the filter. After centrifugation (Centrifuge 5810 R, Eppendorf AG, Hamburg, Germany) with 4,000 rpm, 10 ml of double distilled water was added to the supernatant. This procedure was carried out five times.

##### Size Distribution of PLGA Nanoparticles

The nanoparticle size was determined with dynamic light scattering (DLS) and nanoparticle tracking analysis (NTA). The size measurement of the PLGA nanoparticles was performed by DLS with the Zetasizer Nano ZS (Malvern Instruments, Malvern, United Kingdom) followed by the data processing with the Zetasizer software 6.30. Measurements were performed at 25°C, after an equilibration time of 120 s. All measurements were repeated five times with automatic sub run determination (12–16 runs). All measurements were performed in the 173° backscattering mode. For all DLS measurements the nanoparticle dispersion was diluted to 10% v/v of the purified nanoparticle dispersion with 0.22 μm filtered through a polyethersulfone (PES) filter water.

The NTA experiments were carried using a NanoSight NS300 apparatus (Malvern Instruments, Malvern, United Kingdom). Two different purified AmpB-PLGA ratios (1:10 and 1:20) were investigated. For the nanoparticle tracking performance, the particles were diluted to 1% v/v of the purified particle dispersion with 0.22 μm (PES) filtered water. Five different positions of each particle dispersion were analyzed for 60 s with a 642 nm diode at 25°C. The particle motion was recorded by a sCMOS camera, followed by data processing with the NTA 3.1 software.

#### Drug Load and Entrapment Efficiency of the PLGA Nanoparticles (Ic)

After PLGA nanoparticle (Ic) preparation samples were frozen in liquid nitrogen at −196°C until the whole column was solidified, followed by lyophilization with a Christ Alpha 2-4 freeze dryer (Martin Christ Gefriertrocknungsanlagen GmbH, Osterode am Harz, Germany) for 24 h. A vacuum of 0.06 mbar was applied to the vacuum chamber. Afterwards, lyophilisates were weighed to 10 mg, dissolved in 0.5 ml DMSO and filled up with methanol to 10 ml. Subsequently, the solutions were analyzed by high performance liquid chromatography (HPLC) to determine the PLGA nanoparticle drug load using an Agilent 1100 instrument in combination with an EC 250/4 Nucleodur 100-3 C18ec column. The measurements were performed by an isocratic elution with 60% mobile phase A and 40% phase B. The mobile phase A consisted of methanol and EDTA in water (1.1 g/l) 50/50 whereas the mobile phase B was 100% acetonitrile. A flow rate of 0.7 ml/min was used and temperature of 30°C was applied to the column. AmpB was determined by UV/VIS absorbance at a wavelength of 405 nm. The drug load and entrapment efficiency was calculated by Equations (1) and (2), where m_drug_ is the mass of AmpB, m_sample_ is the mass of the weighed drug containing drug delivery system and m_drug initial_ is the amount of drug which was initially added to the solution for sample preparation.

(1) $$\begin{array}{l} drug\;load\;\left[\%\right]=\frac{m_{drug\;}}{m_{sample}}\;*\;100\% \end{array}$$

(2) $$\begin{array}{l} entrapment\;efficiency\;\left[\%\right]=\frac{m_{drug\;load\;}}{m_{drug\;initial}}\;*\;100\% \end{array}$$

### Amphotericin B-Eudragit L Polyelectrolyte Complex (Id)

#### Preparation of Amphotericin B-Eudragit L Complex (Id)

The preparation of the AmpB complex (AmpB-Eu L) (Id) was carried out by a modified method described previously for AmpB with poly(glutamic acid) (Mohamed-Ahmed et al., 2013). Different AmpB solutions in DMSO were prepared (10, 20, and 30 mg/ml) as well as one Eudragit L solution (Eu-L) with 30 mg/ml prepared in DMSO. Thereafter, 1 ml Eu-L solution was dropped into 1 ml of AmpB solution. The mixture was stirred for 1 h at 600 rpm under light protection. Then, 200 μl of a 1 M aqueous sodium hydroxide was dropped into the AmpB-Eu L solution (1 drop/5 s), followed by dropwise addition of 1 ml 0.2 M sodium hydroxide to the same solution. Subsequently, the solution was dropped into 12.5 ml filtrated water (the water was filtrated through a 0.22 μm PES filter before the experiment). The dilution was stirred again for 1 h at 200 rpm under light protection. To remove residual solvent and non-complexed AmpB, the solution was placed in a dialysis bag (Nadir®-Dialysis Tubing, 10–20 kDa, ø 50 mm, Carl Roth GmbH + Co. KG, Karlsruhe, Germany) and was purified by stirring for 24 h in 3 l distilled water. After the purification step the solution was frozen in liquid nitrogen and freeze dried for 24 h. The lyophilisation process was performed the same way described in section Drug Load and Entrapment Efficiency of the PLGA Nanoparticles (Ic).

#### UV/VIS-Spectroscopy

The UV/VIS spectra were recorded with an UV-1800 spectrometer from Shimadzu (Duisburg, Germany) using a spectral scan from 300 to 450 nm wavelengths. The polyelectrolyte complex (Id), which was prepared from a mass ratio of 3:3, was dissolved in double distilled water, methanol-water 50/50, and AmpB was dissolved in methanol. All samples were diluted with the respective solvent until the absorption was lower than 1.5.

#### Drug Load of Amphotericin B-Eudragit L Polyelectrolyte Complex (Id)

The drug load of the complex (Id) was determined by HPLC as described in section Drug Load and Entrapment Efficiency of the PLGA Nanoparticles (Ic). Therefore, 10 mg of complex was dissolved in 50 ml mixture consisting of double distilled water-methanol (50/50). The drug load was calculated with the following equation, where m_AmpB_ is the mass of the determined AmpB and m_Complex_ is the mass of the weighed polyelectrolyte complex.

(3) $$\begin{array}{l} drug\;load\;\left[\%\right]=\frac{m_{AmpB}}{m_{Complex}}\;*\;100\% \end{array}$$

#### Differential Scanning Calorimetry (DSC)

The thermal behavior of the samples was determined with the differential scanning calorimeter DSC821 from Mettler-Toledo GmbH (Germany). Five milligrams samples were heated from 0 to 240°C with a heat rate of 5 K/min. The cooling curve was recorded by a −10 K/min cooling rate. An empty aluminum pan was used as reference. The measurements were performed under a nitrogen flow of 30–40 ml/min. DSC data were processed by the STAR SW V6.0 software. The polyelectrolyte complex (3:3), Eu-L, AmpB, and the physical mixture were investigated.

#### Heat Induced Stress Test

Investigation of heat influence on the spectral shape of the AmpB complex (Id) was performed by UV/VIS spectral scanning of the complex from 300 to 450 nm wavelengths. A solution with 0.1 mg/ml polyelectrolyte complex (29% AmpB) in double distilled water was prepared. Two milliliters of solution were incubated in a water bath at room temperature, 40, 65 and 80°C for 10 min under light protection, followed by recording the absorption spectra. Furthermore, complex-loaded fibers were dissolved to 0.2 mg/ml in double distilled water and were stored at room temperature, followed by record of the UV/Vis spectra.

#### FTIR-ATR Spectroscopy

Infrared spectra were recorded with a FT-IR spectrometer IFS 28 from Bruker (USA). Measurements were performed with 32 scans and a 2 cm^−1^ resolution. The samples were analyzed on a zinc selenide crystal with 1.3 mm diameter from PIKE Technologies (USA). The angle of incidence and reflection was 45°. The spectra of pure AmpB and Eu-L were recorded. In addition, three different drug-polymer ratios of the polyelectrolyte complex (1:3, 2:3, 3:3) (Id) and a physical mixture (2:3) were analyzed as well.

### X-Ray Powder Diffraction

X-ray powder diffraction was performed with a STOE STADI MP (STOE & Cie GmbH, Darmstadt, Germany). The diffractometer was equipped with a molybdenum anode (50 kV, 30 mA) and a Ge (111) monochromator (Mo K_α_ radiation at 0.071073 nm). All experiments were performed at transmission mode with rotating samples. The transmission mode was performed from 2θ = 5–40° in 0.5° steps. Each step was captured over 60 s using a DECTRIS MYTHEN 1K Strip Detector. The STOE WinXPOW software was used for processing the diffraction patterns. All samples were cryomilled under liquid nitrogen (CryoMill, Retsch GmbH, Haan, Germany) before recording the diffraction diagram.

### Electrospinning

#### Electrospinning of Amphotericin B Free Fibers

The starter kit Spraybase® (Maynooth, Ireland) was used. Gellan Gum LA was weighed and dispersed in double distilled water to a concentration of 0.2% w/v. The dispersion was heated up to 80°C until a clear solution was obtained. Pullulan was added to the hot solution to obtain a concentration of 20% w/v. Afterwards, the solution was treated by a vortex mixer until a homogenous solutions was obtained. Residual air bubbles were removed by centrifugation for 10 min at 1,000 rpm (Labofuge 300, Kendro, Germany). The polymer solution was electrospun with a flow rate of 0.75 ml/h, a 22 G needle and needle-collector distance of 10 cm. The voltage of the process was increased until a stable Taylor cone was obtained. Depending on the spinning solution, a voltage between 12 and 14 kV was applied (Göttel et al., 2020). All solutions were electrospun with the described parameters.

#### Preparation of Amphotericin B Loaded Fibers

Five milligrams AmpB was weighed in a glass vessel and 4 ml of double distilled water was added to the drug. The dispersion was stirred under light protection for 1 h at 360 rpm with a magnetic stirrer. Afterwards, the dispersion was centrifuged to get the supernatant. The supernatant was used for preparation of a solution containing 0.2% Gellan Gum LA and 20% Pullulan. The solution was processed as described above (IIa). To increase the AmpB drug load of the fibers, the AmpB dispersion was produced with double distilled water containing also 1 mg/ml sodium cholate. The received supernatant was processed as stated above (IIb).

#### Amphotericin B-PLGA Nanoparticle Loaded Fibers (IIc)

Ten milliliters of the purified AmpB-loaded PLGA nanoparticles (Ic) with the ratio 2:10 were concentrated with the Amicon® Ultra 15 ml centrifugal filter. After that, the particle dispersion was filled up to 3 ml with distilled water. A spinning presolution containing 26% w/v Pullulan and 0.3% w/v Gellan Gum was prepared. The solution was filled up with the nanoparticle dispersion to 20% Pullulan and 0.2% Gellan Gum in aqueous phase. After homogenization air bubbles were removed by centrifugation and the solution were electrospun (IIc).

### Polyelectrolyte Complex Loaded Fibers (IId)

A polyelectrolyte complex (Id) (3:3) solution with 20 mg/ml was prepared in double distilled water. 0.5 ml of the solution was added to a hydrated 0.3% w/v Gellan Gum solution containing 26% w/v Pullulan, so that a concentration of 20% Pullulan and 0.2% Gellan Gum were obtained.

#### Fiber Size Distribution

The fiber morphology and size distribution of the fibers were determined by the ESEM XL 30 FEG (Philips Electronic Instruments, Mahwah, U.S.) with a Gaseous Secondary Electron detector (GSE). To avoid electrical charge of the fibers, the images were captured at 0.9 Torr and an accelerating voltage of 12 kV. Fiber size was determined by the IC Measure® software.

### Drug Load of Electospun Nanofibers

Thirty milligrams fibers (IIa-IIc) were dissolved in 1 ml DMSO. One milliliter of each solution was diluted to 10 ml with methanol. The solution was centrifuged for 10 min with 6,000 rpm (IKA Mini G, IKA®-Werke GmbH & CO. KG, Staufen, Germany).

The drug load of the complex-loaded fibers (IId) was determined by weighing 10 mg fibers (IId), followed by dissolving in 2 ml double distilled water. 0.5 ml solution was diluted with methanol to 1 ml. The dilution was centrifuged to remove precipitating polymer. The obtained supernatants were analyzed by HPLC as described in section Drug Load and Entrapment Efficiency of the PLGA Nanoparticles (Ic).

### Plate Diffusion Test: *Issatchenkia orientalis*

Antimycotic properties of the different AmpB-loaded nanofibers were investigated by a plate diffusion test with *Issatchenkia orientalis* (Candida krusei, Leibnitz-Institute DSMZ German Collection of Microorganisms and Cell Cultures GmbH, Braunschweig, Germany). The freeze dried strain was treated with 0.5 ml of autoclaved universal medium for yeasts (3 g yeast extract, 3 g malt extract, 5 g peptone form soybeans, 10 g glucose, 15 g agar, 1 l double distilled water). After 30 min of swelling, 0.25 ml fungal suspension was placed in 5 ml of the described yeast medium and incubated for 24 h in End-Over-End mixer at 25°C under light protection. After 24 h, 0.25 ml cell dispersion were seeded on an agar containing yeast in petri dishes.

Different AmpB formulations were tested. AmpB was dissolved in a 2% DMSO-methanol mixture to 100 μg/ml concentration. Ten microliters containing AmpB solution and 10 μl of pure solvent mixture were placed on different petri dishes. Electrospun disks with 4 mg weight and 1.5 cm diameter of different nanofibers were analyzed as well. Drug-free Pullulan-Gellan Gum nanofibers containing sodium cholate, AmpB-sodium cholate loaded fibers (IIb) and AmpB-Eu L complex loaded fibers (IId) were investigated. The electrospun disks were hydrated by residual moisture at the petri dish without further addition of any hydrating agents. After sample instillation, the petri dishes were incubated at 25°C for 24 h under light protection, followed by analyzing the inhibitory region without quantification.

#### Zeta Potential of the Amphotericin B Loaded PLGA Nanoparticles (Ic) and AmpB-Eu L Complex (Id)

The zeta potential was measured with the Zetasizer Nano ZS (Malvern Instruments, Malvern, United Kingdom) using laser Doppler electrophoresis. PBS and STF were diluted 1:99 and the pH of both buffers was adjusted to 7.4. The purified nanoparticle dispersion (Ic) was diluted (1:9) with the diluted buffer solutions. To determine the zeta potential of the complex (Id), solutions with 5 mg/ml in double distilled water were prepared, followed by dilution to 0.5 mg/ml with each buffer. Different AmpB and Eu-L ratios (1:3, 2:3, 3:3) were investigated. The obtained solutions were characterized immediately after preparation. All samples were equilibrated for 120 s to 25°C. The numbers of performed sub runs were optimized by the Zetasizer software. Each sample was measured in quintuple. The data were processed by the Zetasizer software 6.3.

### Cytotoxicity of Multilayer-Stratisfied Human Epithelium Cells

For cell culture 17,000 htCEpi (Immortalized human corneal epithililal cell line, Evercyte, Austria) cells were seeded into 24 well inserts with a pore size of 0.4 μm. The cells were incubated for 7 days in culture media. Before 1.15 mM calcium chloride^*^2 H_2_O was added to the culture media. The KGM-2 Bullet kit (Lonza, Switzerland) was used as differentation medium. After 14 days of incubation as an air lifted model, a multilayered cornea model was obtained differentiated into basal cells, wing cells and superficial cells. At day 21 40 μl of 0.62 μg/ml Amphotericin B eye drops diluted with 0.9% sodium chloride (Amphotericin B, 50 mg, Bristol-Meyer-Squibb), 5 mm slices of blank fibers and AmpB-Eu L complex loaded fibers were placed onto the air lifted side of the cells and were incubated for 1 day. After 1 day immunocytochemical assay with KI-67 antigen as a proliferation marker was performed, followed by microscopic determination of the cellular relative viability inside the wing and basal cell layer.

### Impact of Electron Beam Sterilization

All samples were treated with 25 kGy electron beam at room temperature. The electron beam was performed by a linear accelerator MB 10-30 MP (Mevex, Stittsville, Ontario, Canada) at 10 MeV of the Leibnitz Institute of Surface Engineering (Leipzig, Germany). The repetition rate of the accelerator was 460 Hz with 8 μs pulses, using a scanning frequency of 3 Hz and a scanning width up to 60 cm. The dose was determined with a graphite calorimeter with an error of 5%.

Potential degradation of Pullulan and Gellan Gum was investigated by asymmetric flow field-flow fractionation (A4F). The A4F instrument (Eclipse 3+, Wyatt Technology Europe, Dernbach, Germany) was connected to an isocratic pump (Aligent 1200 series), an AF4 channel (length 153 mm, largest width 21 mm, nominal height 350 μm; PES membrane MWCO 10 kDa), a multi-angle laser light scattering detector (DAWN Heleos II) and a differential refractive index detector (Optilab rEX, all from Wyatt). Two hundred microliters sample (2 mg/ml polymer in purified water preserved with 0.02% sodium azide) were injected (0.2 ml/min) in the focus mode (focus flow 2 ml/min, total focusing time 5 min) and then eluted at a detector flow of 1 ml/min applying the following cross flow conditions: Cross flow decreasing from 2.0 to 0.1 ml/min over 30 min and a constant cross flow of 0.1 ml/min for 5 min followed by elution without applied cross flow. Preserved (0.02% sodium azide) and filtered (pore size 0.1 μm) purified water served as carrier liquid. For molar mass determination, the light scattering signals were fitted with the Debye (Pullulan) or random coil (Gellan Gum) fit model using a dn/dc of 0.146 and 0.135 for Pullulan and Gellan Gum, respectively (Astra 6 software, Wyatt).

Furthermore, the drug before and after sterilization of pure AmpB, Eu-L complex (Id) and the complex-loaded fibers (IId) were determined by HPLC as described in section Drug Load of Amphotericin B-Eudragit L Polyelectrolyte Complex (Id).

## Results and Discussion

### Characterization of PLGA Nanoparticles (Ic)

**Figure 2:**
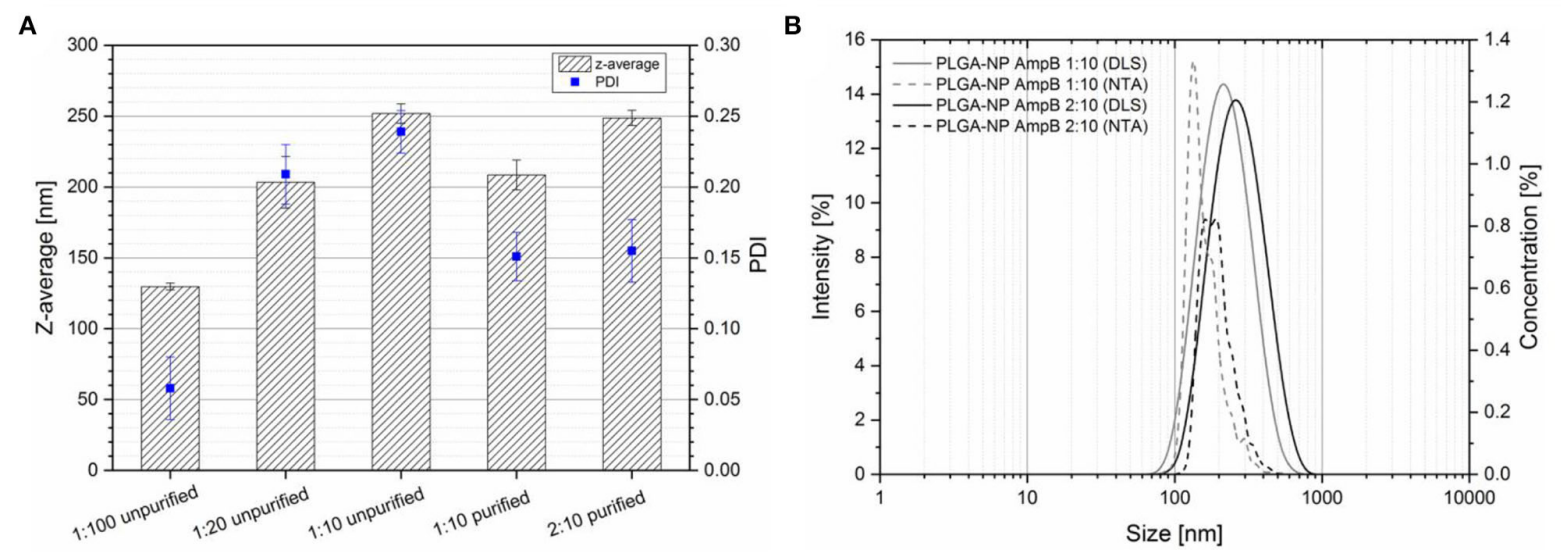
**(A)** Particle size and PDI (obtained by dynamic light scattering) of different PLGA-AmpB ratios before and after centrifugal purification steps (mean ± SD, *n* = 3). **(B)** Nanoparticle size distribution of different purified PLGA-AmpB nanoparticle formulations determined with DLS (solid lines) and NTA (dashed lines).

The results of the DLS measurements are displayed in Figure 2A. Three different ratios of AmpB and PLGA at 1:100, 1:20, and 1:10 were tested and analyzed to obtain monodisperse nanoparticles without the previous described purification steps. The z-average diameter at the ratio of 1:100 was 129.8 nm with a PDI of 0.058. The ratio of 1:20 had a diameter of 203.4 nm and a PDI of 0.209 and the ratio of 1:10 had a z-average of 251.9 nm and a PDI of 0.239. Hence, increasing the amount of drug in the polymer led to bigger particles with an increased PDI. After the purification steps, the z-average of the 1:10 nanoparticles was 208.6 nm with a PDI of 0.151 and the 2:10 ratio had a diameter of 248.8 nm and a PDI of 0.155. The z-average and the PDI of the formulation decreased after removing DMSO by centrifugation.

To stabilize the nanoparticle dispersion, surface active ingredients are necessary to avoid particle aggregation. During the nanoparticle preparation, AmpB was able to interfere with PVA at the particle surface. As a consequence, the particle size increased, caused by hindering the formation of small liquid droplets during the injection process. Furthermore, AmpB interacted with PVA at the water-polymer interface which resulted in an increase of the PDI. After purification, the z-average and the PDI of the formulations decreased because non-entrapped AmpB and the free PVA were removed from the system.

To gain a deeper insight into the size distribution, two different methods (DLS and NTA) were used to investigate two different AmpB-PLGA ratios (Figure 2B). The gray curves show the data of the 1:10 ratio and the black ones of the 2:10 ratio, while the solid lines represent the results of DLS and the dashed lines the results of the NTA measurements. Both methods showed a distinct shift of the particle size to higher sizes with increasing drug content. The mean size of the NTA measurement was 179.5 nm diameter for the 1:10 and 213 nm for the 2:10 ratio. The z-average measured by DLS was 208.6 nm for the 1:10 ratio and 248.8 nm for the 2:10 ratio. The differences of the determined diameters were caused by size calculation of each method. For DLS, the intensity weighed hydrodynamic radius was used, where particles with higher size contribute much more to the signal intensity (I ~ d^6^). The NTA analysis based on the size calculation from the Brownian motion of the particles. Larger particles move more slowly than smaller ones. The size was calculated by the moved distance of the particles during a defined time period, so that the particle size can be analyzed independently from scattering phenomena compared to DLS (Filipe et al., 2010). In contrast to DLS, the NTA analyzed the size of every single particle what allowed a deeper insight into the particle size distribution. Due to the fact that larger particles scatter the light more intense than smaller ones, the z-average was shifted to higher values by DLS. The NTA results showed that the distribution width increased with increasing the amount of initial added drug, which correlated with the increased PDI of the DLS experiments. In Table 1, the results of the nanoparticle characterization are summarized. The drug load of the purified nanoparticle formulations increased with the initial added drug from 3.9 to 7.2%. Entrapment efficiency showed no significant difference between both drug-polymer ratios with 40.15% (1:10) and 37.09% (2:10). Because of the amphiphilic nature of AmpB, only moderate encapsulation efficacy values of about 40% were obtained.

**Table 1:** Results of NTA and DLS experiments: Drug load, entrapment efficiency, z-average, and PDI of the AmpB loaded PLGA nanoparticles (mean ± SD).

**AmpB-PLGA ratio**	**Drug load [%]**	**Entrapment efficiency [%]**	**z-average [nm]**	**PDI**	**Mean size [nm] (NTA)**
1:10	3.9 ± 0.47	40.15 ± 2.15	208.6 ± 10.5	0.151 ± 0.017	179.5 ± 7.5
2:10	7.2 ± 0.58	37.09 ± 5.09	248.8 ± 5.3	0.155 ± 0.022	213.2 ± 18.7

### Characterization of AmpB-Eu-L Polyelectrolyte Complex (Id)

**Figure 3:**
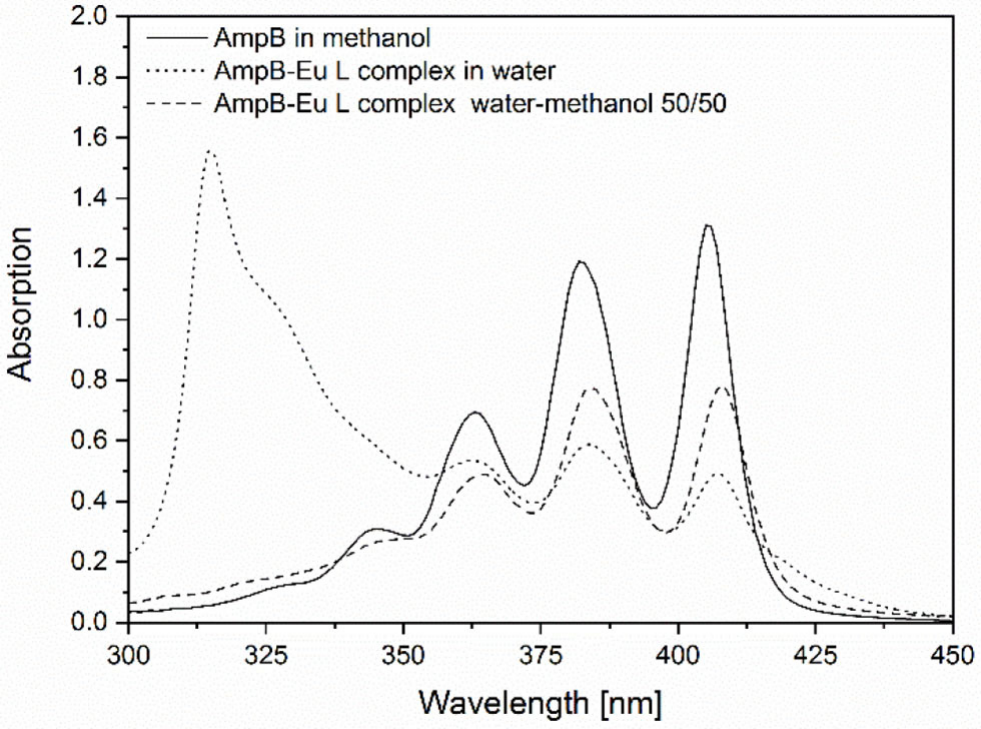
UV/Vis spectra of AmpB in methanol, AmpB-Eu L polyelectrolyte complex (Id) (3:3) in water and in a water-methanol mixture 50% v/v.

Figure 3 shows the UV/Vis spectra of AmpB in methanol, AmpB-Eu-L complex (Id) in water and in a mixture of methanol-water mixture 50% v/v. The solid curve is the characteristic spectrum for monomeric AmpB (Espada et al., 2008). The spectrum is characterized by four different peaks at 405, 382, 363, and 345 nm. After complex formation between AmpB and Eu-L, the spectra changed to a spectrum with an absorption maximum at 316 nm in water (dotted line). After addition of 50% methanol the spectra changed again to the monomer spectra of AmpB (dashed line).

Comparing the spectra of the monomeric AmpB and the complex (Id), the spectra illustrated, that the drug bound to the polymer. After addition of methanol the bounded drug was released from the polymer chain interaction into monomeric form. Similar results are described in the literature with AmpB and poly(α-glutamic acid) after polyelectrolyte complex formation (Mohamed-Ahmed et al., 2013). Caused by reduced electrostatic forces between the positive loaded ammonium group of AmpB and the negative loaded carboxylic function of Eu-L, the drug converted into the monomeric form.

**Figure 4:**
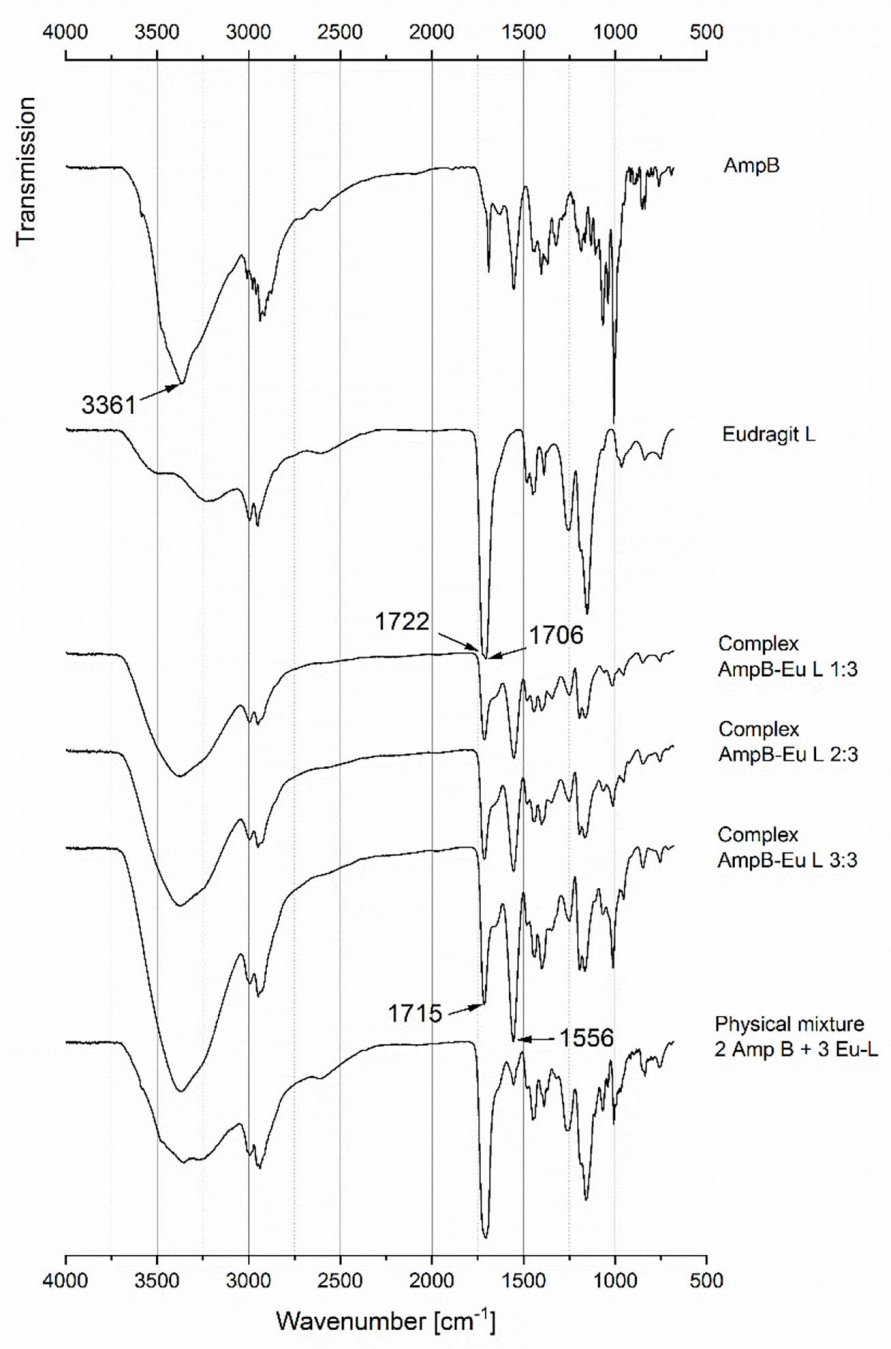
ATR-IR spectra of AmpB, Eu-L, AmpB-Eu L complexes (Id) and a physical mixture.

To gain more information about the chemical structure of the complex (Id) the ATR-IR, spectra of pure AmpB and Eu-L were recorded. Additionally, three different complex ratios and a physical mixture were investigated (Figure 4). The vibration at 3,361 cm^−1^ of AmpB was caused by the NH_2_ functional group. The stretching of Eu-L at 1,722 and 1,706 cm^−1^ was caused by the carbonylic ester and the carboxylic group. In comparison to the physical mixture, the complex showed significant different bands at 1,715 and 1,556 cm^−1^. Similar results were published, there is discussed, that the new existing band at 1,556 cm^−1^ results from the ionic interaction of the protonated ${\text{NH}}_{3}^{+}$ and the deprotonated COO^−^ (Moustafine et al., 2008). The authors describe a non-covalent complex of Eu-L and chitosan. Chitosan as well as AmpB are characterized by primary amino groups, which become protonated at neutral pH. Thereafter, they are able to form ionic interactions between negatively charged carboxylic groups. Figure 4 illustrates that with an increase of the AmpB content, the extent of the band at 1,556 cm^−1^ increased as well. The transmissions at 1,715 and 1,556 cm^−1^ of the physical mixture were lower in comparison to the band of the polyelectrolyte complex (Id).

The drug load of three different complex (Id) formulations was determined by HPLC. The results are displayed in Table 2. With an increasing amount of added AmpB, the drug load of the complex increased from 3.8to 11.0 to 28.9%. All further preparations of polyelectrolyte complex loaded fibers (IId) were performed with the formulation containing 28.9% AmpB (Id).

**Table 2:** Drug load of different of polylectrolyte complex prepared with different Amphotericin B and Eudragit L ratios (mean ± SD).

**Amphotericin B**	**Eudragit L**	**Drug load [%]**
1	3	3.83 ± 0.16
2	3	11.02 ± 0.36
3	3	28.91 ± 0.70

**Figure 5:**
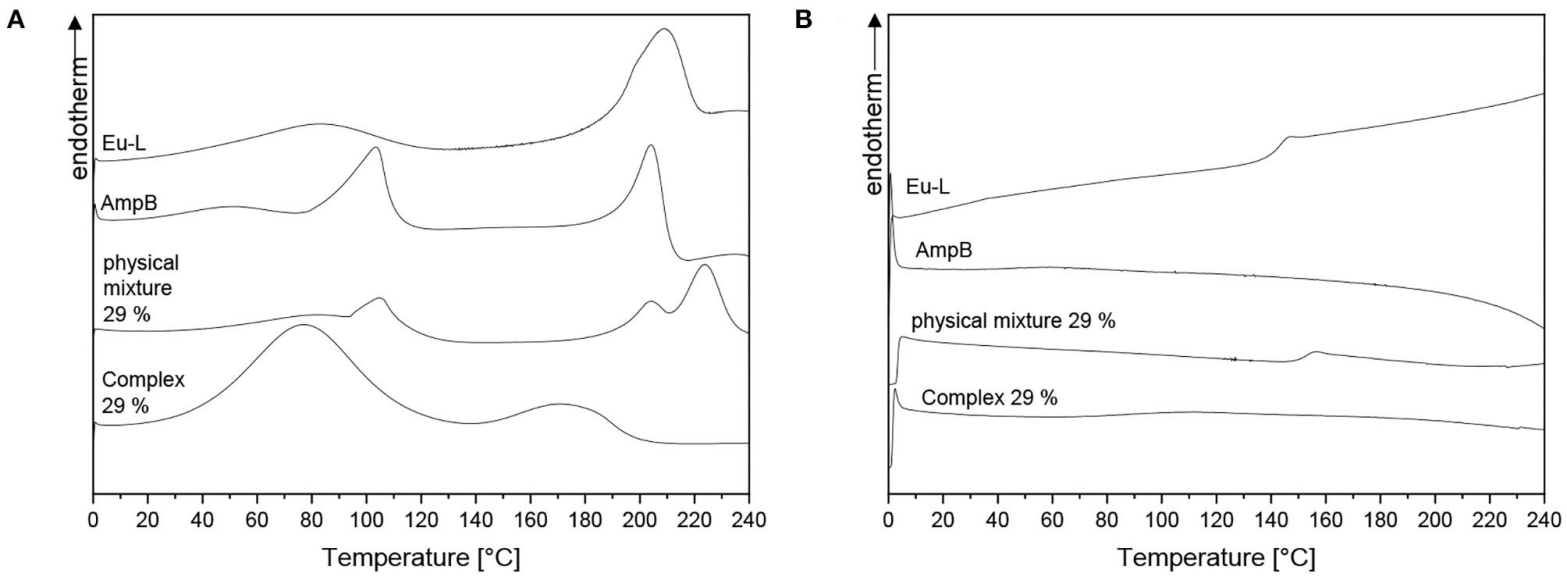
DSC measurements of pure Eu-L, AmpB, AmpB-Eu L polyelectrolyte complex (Id), physical mixture: **(A)** First heating curve; **(B)** Second heating curve.

The first and the second heating curves of the DSC measurements are displayed in Figures 5A,B, respectively. The first heating curve (A) of Eu-L showed two endotherm peaks, one at 83°C and another one at 208°C. The second heating curve (B) Eu-L showed a glass transition at 143°C. During the first heating cycle, AmpB had an endotherm peak at 103 and 204°C, which are also described in the literature (Angra et al., 2009; Ghosh et al., 2017). It is well-known that AmpB starts to degrade at temperatures over 200°C without melting (Kim et al., 2010). The peak at 204°C indicated the heat induced degradation of the drug. The degradation was concluded by the absence of any thermal effect in the second heating curve (B). The physical mixture showed three different endotherm peaks at 103, 204, and 224°C. The peaks at 103 and 204°C were caused by AmpB and the peak at 224°C was caused by the Eu-L. After the first heating cycle the physical mixture showed a glass transition at 153°C caused by Eu-L (Figure 5B).

In contrast to pure AmpB and Eu-L, the polyelectrolyte complex (Id) showed two broad endotherm peaks, one at 77°C and the other one at 174°C. Both peaks were not comparable to the endotherm phenomena of the pure substances. For the peak at 77°C, we postulate that water started evaporation from the sample. During the second heating cycle, no thermal effects of the complex (Id) were detected. The complex decomposition at 170°C seemed to be supposable because of the absence of the glass transition of Eu-L.

**Figure 6:**
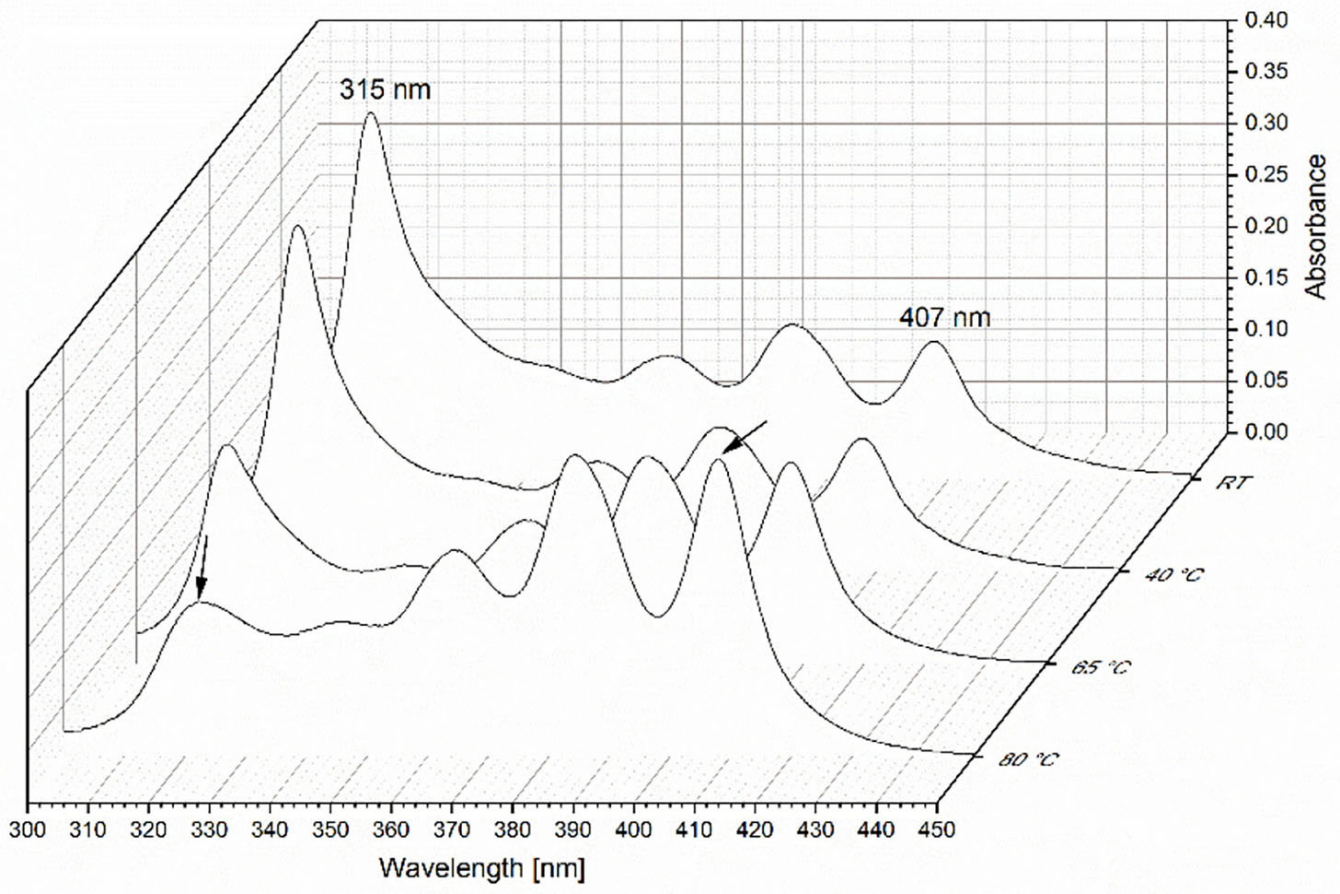
UV/Vis spectra of AmpB-Eu L polyelectrolyte complex (Id) at room temperature (RT), 40, 65, and 80°C after incubation time of 10 min.

In Figure 6, the results from the heat induced stress test are displayed. The UV/Vis spectra of the complex (Id) after treatment with four different temperatures are compared. The spectrum of the complex stored at room temperature showed no difference to the complex spectrum in water in Figure 3. The absorbance maximum was determined at 315 nm. After incubation at 40°C no distinct differences were determined compared to the sample spectra stored at room temperature. In contrast, incubation at 65 and 80°C resulted in a decreased absorbance at 315 nm. Furthermore, the absorbance at 407 nm increased. The spectra of the complex changed to that of the monomeric AmpB (see Figure 3). We observed that after heating at 65°C the AmpB-Eu L complex (Id) starts dissociation, so that the drug is available in the monomeric form.

### X-Ray Powder Diffraction

**Figure 7:**
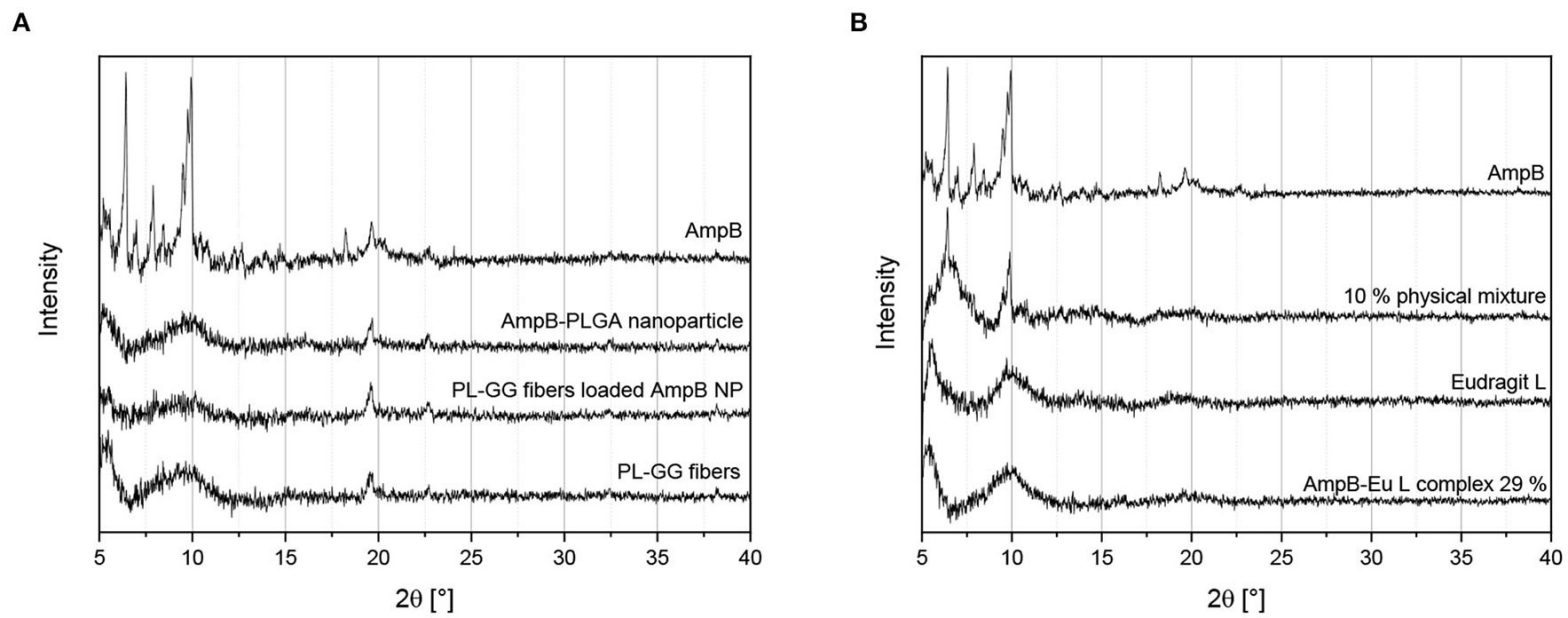
X-ray powder diffraction patterns of different samples: **(A)** AmpB loaded PLGA nanoparticles (Ic), unloaded nanoparticles, electospun Gellan Gum-Pullan fibers (PL-GG fibers) and PLGA nanoparticle loaded fibers (IIc) containing AmpB; **(B)** AmpB, physical mixture of 10% AmpB in Eu-L, Eudragit L and 29% polyelectrolyte complex (Id) (AmpB-Eu L complex 29%).

Figure 7A represents the diffraction patterns of AmpB, AmpB-loaded PLGA nanoparticles (Ic), AmpB-PLGA-loaded Pullan-Gellan Gum fibers (IIc) and the blank fibers. AmpB showed clear reflections at 6, 10, 18, and 24°. The AmpB-loaded nanoparticles (Ic) showed in contrast to AmpB no additional reflections to the investigated polymers. With the entrapment of AmpB into the PLGA nanoparticles, the crystalline nature of drug disappeared and a molecular disperse AmpB distribution inside the particles was obtained. The diffractograms of the nanoparticle-loaded fibers (IIc) as well as the blank fibers did not show reflections due to their amorphous nature.

Figure 7B depicts the diffraction diagram of pure AmpB, 10% w/w physical mixture of AmpB and Eu-L, pure Eu-L, and the polyelctrolyte complex (Id). Pure AmpB as well as the physical mixture of the drug-polymer showed the main reflexes of AmpB at 6° and 10°. In contrast to the physical mixture, Eu-L and the polyelectrolyte complex (Id) showed no diffraction patterns. During the complex preparation, the crystalline AmpB was changed to an amorphous substance by binding to Eu-L. Followed by complex (Id) formation, the distance between the AmpB molecules might have increased and with increasing distance, lattice formation of the AmpB molecules was prevented.

### Zeta Potential of PLGA Nanoparticles (Ic) and AmpB-Eu L Polyelectrolyte Complex (Id)

**Figure 8:**
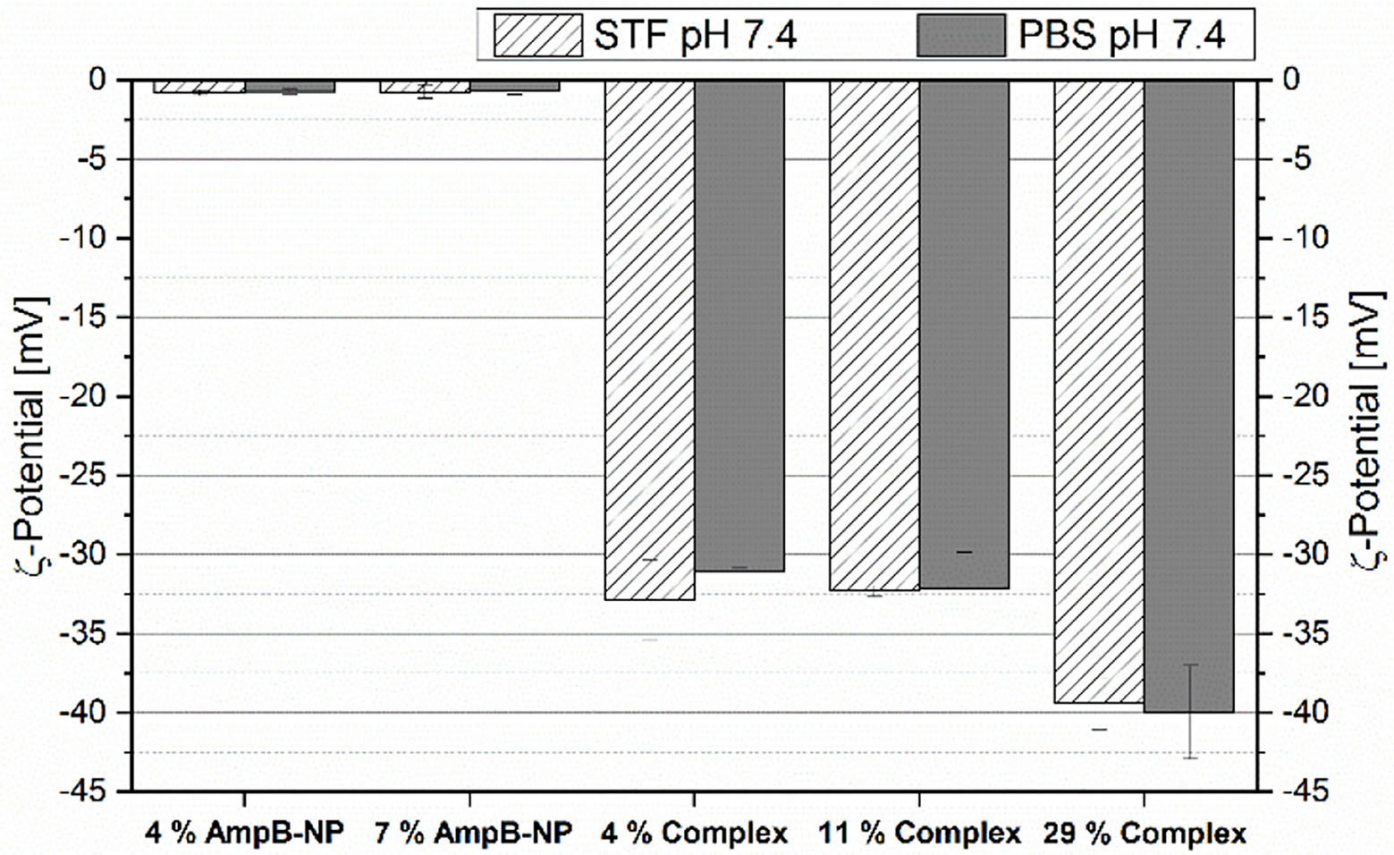
Zeta potential of different AmpB loaded PLGA nanoparticles (Ic) (4%, 7%) and different AmpB-Eu L complexes (Id) (4, 11, and 29%) in PBS pH 7.4 and STF pH 7.4 (mean ± SD, *n* = 3).

Figure 8 displays the results from the zeta potential measurements of the PLGA nanoparticles (Ic) (1:10 and 2:10) and differently loaded AmpB-Eu L complexes (Id). The zeta potential of the AmpB-loaded nanoparticles (Ic) showed no distinct difference between the two drug/polymer ratios and the used buffer. The surface charges of both particle formulations were nearly neutral. In comparison to the particles, the complex showed a negative surface. The 4% and the 11% loaded complexes (Id) showed with −32 mV no distinct change of the zeta potential with increasing AmpB content. The complex (Id) containing 29% AmpB had a potential of −39 mV and seemed to be slightly more negative. No distinct differences between the buffers (PBS and STF at pH 7.4) were detected.

The low nanoparticle (Ic) potential of −0.7 mV was caused by the used PVA (Van de Ven et al., 2012). PVA as a stabilizing agent for nanoparticle preparation is known for its isolating properties. In contrast to the PLGA nanoparticles (Ic), the complex (Id) was more negatively charged. Eu-L is a copolymer containing methacrylic acid and acrylic esters. During the complex (Id) preparation, the pH of the polymer solution shifted above 7. As a consequence of the alkaline pH, the carboxylic groups of the polymer became deprotonated and negatively charged. During addition of AmpB, the pH became neutralized. Thereby, the amino groups of AmpB became protonated and bound the negative carboxylic groups by ionic interaction. Furthermore, AmpB contains a carboxylic group which was deprotonated at pH 7.4 and let the surface charge of the complex (Id) decrease.

### Electrospinning and Characterization of Different Fiber Formulations: Morphology and Drug Load

**Figure 9:**
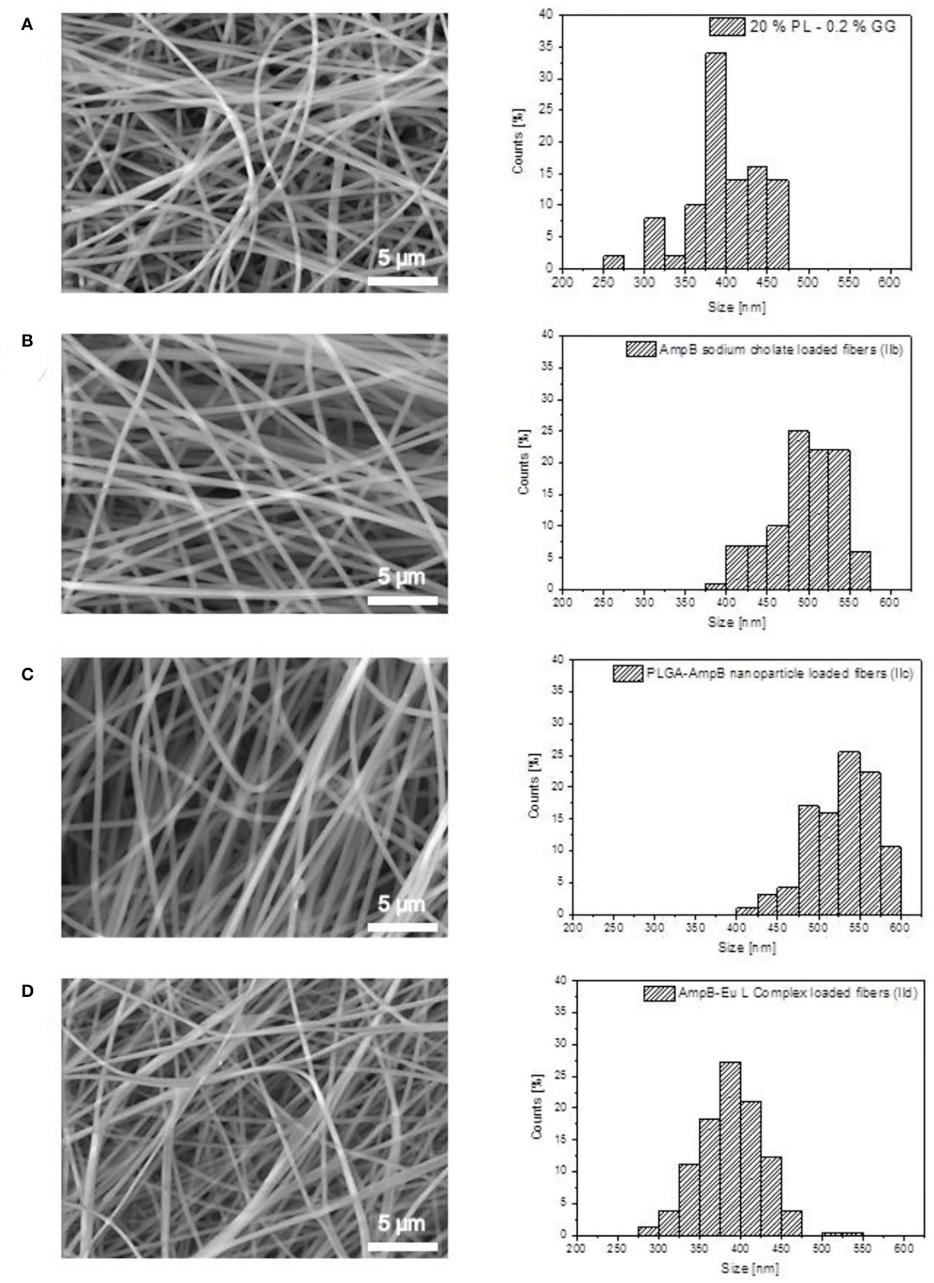
ESEM images and fiber size distribution of Pullulan-Gellan Gum nanofibers: **(A)** Unloaded fibers; **(B)** Sodium cholate-AmpB-loaded fibers (IIb); **(C)** PLGA-AmpB nanoparticle-loaded fibers (IIc); **(D)** AmpB-Eu L polyelectrolyte complex (29%)-loaded fibers (IId).

The morphology and fiber diameter of different drug load formulations were analyzed by ESEM (Figure 9). All spun fibers were homogenous and free from defects. Figure 9A shows the data of the blank Pullulan-Gellan Gum fibers. The fiber main fraction was 375–400 nm (Göttel et al., 2020). The mean fiber diameter of AmpB sodium cholate-loaded fibers (IIb) increased to 475–500 nm (Figure 9B), to 525–550 nm for the AmpB-PLGA nanoparticle-loaded fibers (IIc) (Figure 9C) and to 375–400 nm for the AmpB-Eu L complex (IId) (29%, Figure 9D).

All formulations were appropriate for electrospinning and resulted in homogenous fiber morphology with sizes around 400–600 nm. After addition of AmpB and sodium cholate to the Pullan-Gellan Gum formulation (IIb), the fiber diameter increased. Sodium cholate as additional excipient add sodium cations to the spinning solution. In eye drop formulations, Gellan Gum is well-known as an *in situ* gelling agent increasing the solution viscosity and ocular bioviability. The carboxylic groups of the polymer are able to form gels with mono- as divalent cations. After addition of sodium cholate, the carboxylic groups of the polymer started to interact with the sodium cations. Therefore, the viscosity of the spinning solution increased and caused fiber diameter increase (Deitzel et al., 2001). On the other hand, the addition of sodium cholate to the spinning solution, increases the conductivity and affects the spinnability of the system (Uyar and Besenbacher, 2008).

In comparison to the blank spun fibers, the nanoparticle loaded fibers (IIc) showed a higher fiber diameter as well. During the nanoparticle preparation (Ic), PVA was added to the aqueous phase to stabilize the formulation. Excess PVA was washed out by the purification steps as described above, but the PVA localized at the nanoparticle surface was able to expand the interaction between the Pullan, Gellan Gum and the nanoparticles (Ic). The hydroxylic groups of PVA were able to form hydrogen bonds to polymers, caused by the increased interaction of the Pullulan, Gellan Gum and PVA, as well as the higher total amount of polymer in the spinning solution enabled the preparation of fibers with larger diameters.

By electrospinning of the AmpB-Eu L complex (Id) in the Pullulan-Gellan Gum solution, the fiber diameter (IId) showed no significant difference in size to the pure blank fibers. One reason might be the addition of the polyelectrolyte complex combined with the conductivity increase. The addition of the polyelectrolyte complex (Id) may increase the solution viscosity by a higher amount of polymer inside the spinning solution, but the increase of the fiber diameter caused by the higher viscosity becomes compensated by the increased conductivity of the solution.

**Figure 10:**
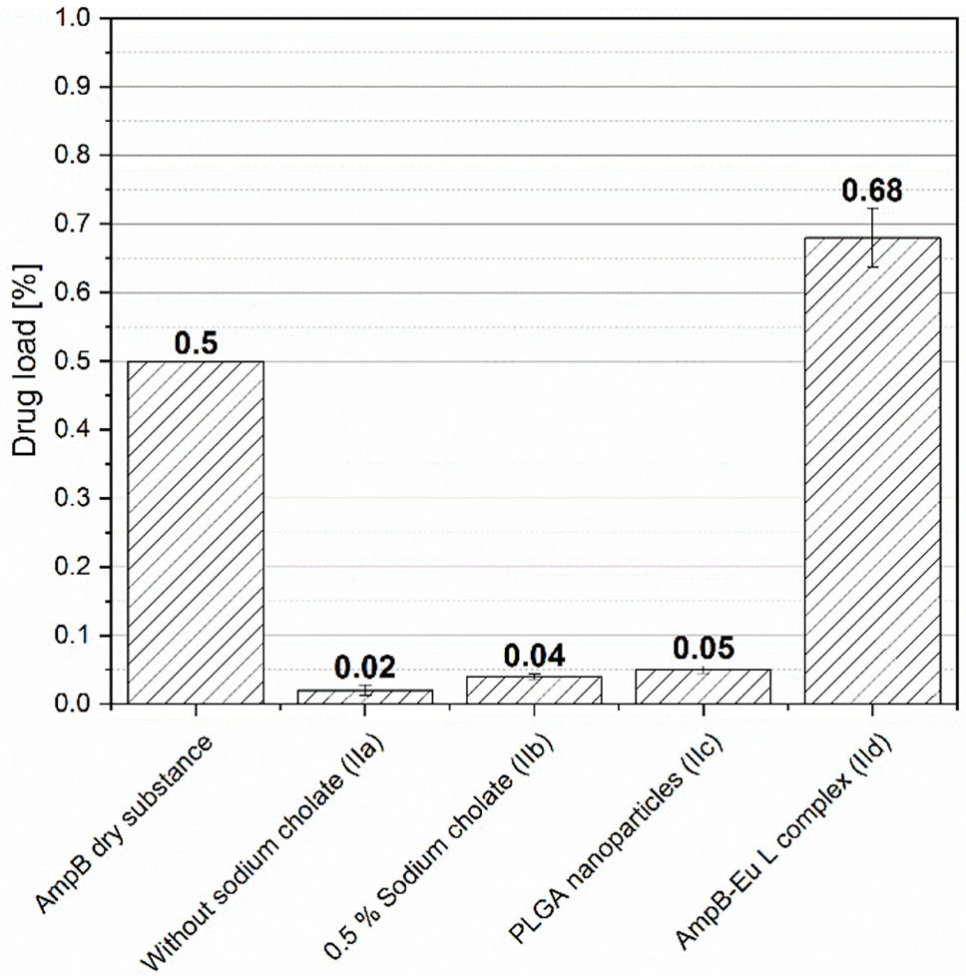
Amphotericin B drug load of different Pullulan-Gellan Gum nanofiber formulations (IIa-IId). AmpB dry substance illustrates the drug load of the used eye drops in practice (mean ± SD, *n* = 3).

Figure 10 displays the drug load of different formulations. Patients with keratomycosis are often treated with aqueous AmpB eye drops. Different AmpB concentrations from 0.15 to 0.7% are used frequently in combination with triazole antimycotics as subconjunctival injection for the antifungal therapy (Díaz-Valle et al., 2002; Bourcier et al., 2003; Mahdy et al., 2010). The AmpB loaded fibers without sodium cholate (IIa) had a concentration of 0.02% AmpB. After addition of 0.5% sodium cholate as amount of fiber matrix to the AmpB solution the content of the fibers IIb increased to 0.04%. During the incorporation of the AmpB loaded PLGA nanoparticles (IIc), a drug content of 0.05% was attained. A more efficient AmpB drug load by addition of sodium cholate (IIb) and by encapsulation of AmpB in nanoparticles (IIc) was thus not achieved. The formulations with pure AmpB (IIa), sodium cholate (IIb) and drug incorporation into nanoparticles (IIc) showed no significant increase of the fiber drug load. Compared to conventional AmpB eye drops, the drug concentration was 10-fold lower, which might be to low for a practical use despite an anticipated prolonged residence time.

In Figure 10, the addition of AmpB-Eu L polyelectolyte complex (Id) to the formulation reached a drug load of 0.68% for the fibers IId, which is comparable to the load of commonly used eye drops in clinical practice. As described above, the AmpB load of the fibers is limited by the water solubility of the drug < 1 mg/l (pH 6–7) (Lemke et al., 2005). Caused by the amphiphilic nature and the low water solubility further ingredients like bile salts or phospholipids are necessary to increase the dose in aqueous media.

Bile salts (Ib and IIb) are powerful solubilizing agents, but they have a high cytotoxic potential (Dangi et al., 1998; Furrer et al., 2000, 2002). As consequence, we did not add sodium cholate to the spinning solutions in concentrations higher than 0.5% (m/m) into the fiber material to avoid toxic effects at the ocular surface. Caused by the low drug load, the formulations with bile salt and nanoparticle addition were excluded from further experiments. Instead, we focused our work on the complex loaded fibers (IId).

### Plate Diffusion Test: *Issatchenkia orientalis*

The amount of AmpB which can be applied for antifungal therapy is restricted by the lens weight. The weight of the investigated dissolving lens was limited to 4 mg for the following reasons: Lenses with weight greater than 4 mg with a diameter of 15 mm resulted in gel thicknesses which were sheared off from cornea by the lid blink in pre-experiments. Therefore, we investigated lens geometries only, which are meaningful for a practical use.

**Figure 11:**
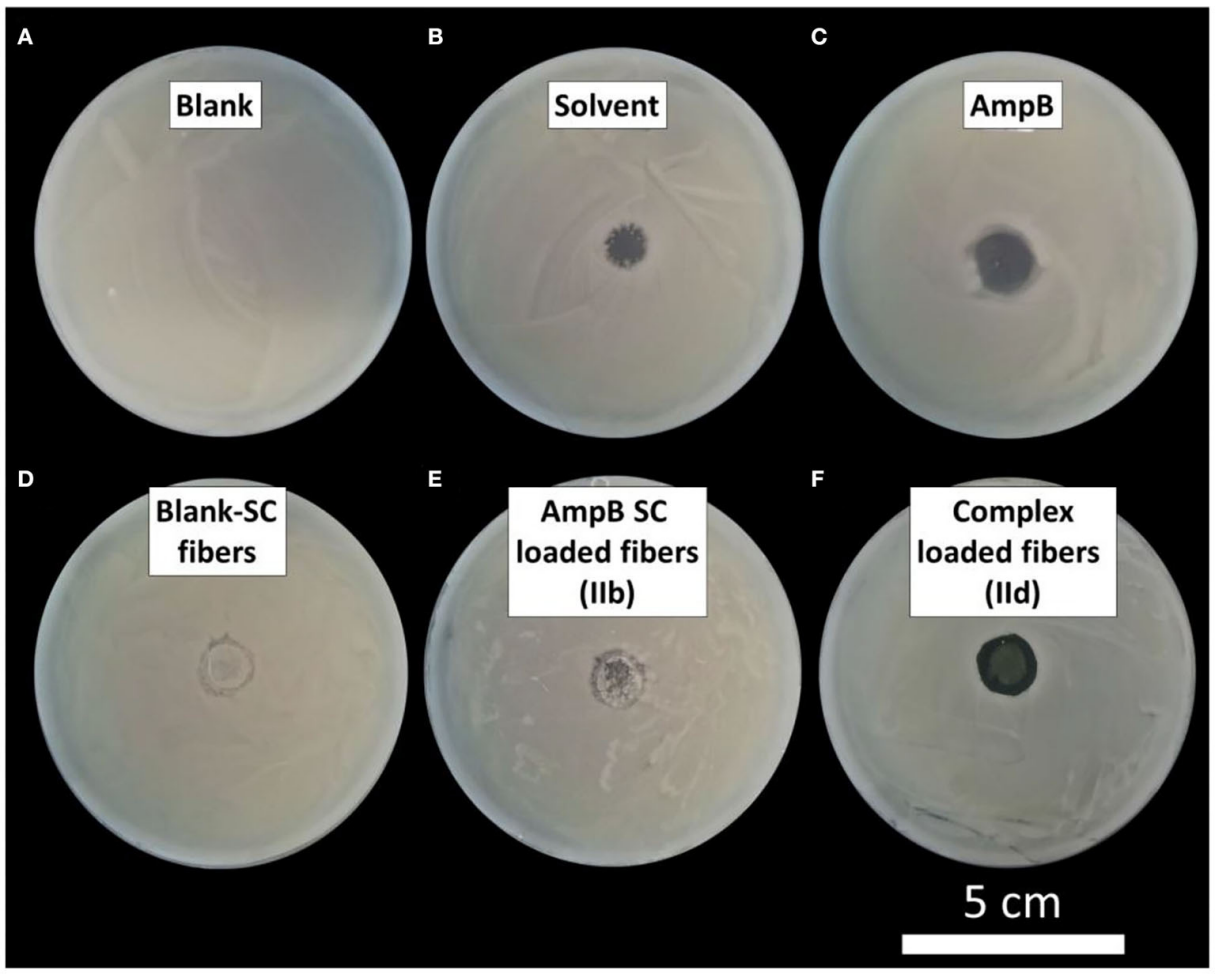
Plate diffusion test of different formulations: **(A)** Blank petri dish; **(B)** 10 μl 2% DMSO-methanol (solvent); **(C)** 10 μl AmpB in 2% DMSO-methanol; **(D)** Blank Pullulan-Gellan Gum-sodium cholate nanofibers (Blank-SC); **(E)** AmpB-sodium cholate loaded fibers (IIb); **(F)** Polyelectolyte complex loaded fibers (IId).

In Figure 11, the images of the incubated plate diffusion tests are displayed. Based on the low solubility of AmpB in water, the drug was dissolved in a mixture of DMSO and methanol. Because of the toxicity of the solvent, the pure solvent mixture was investigated as a reference. The inhibitory zone of the drug (Figure 11C) was larger than the zone of the pure solvent (Figure 11B). Blank fibers showed a very small inhibition zone (Figure 11D). Reasons for that could be the small amount of the incorporated sodium cholate inhibited the fungal growth caused by the high cytotoxicity. On the other hand, a volume contraction after placement of the dry lens onto the agar took place. Hence, regions without cell suspension around the applied lens were induced by the hydration process. The incorporation of AmpB (IIb) showed an inhomogeneous region without a growth of the fungi (Figure 11E). As consequence, the amount of incorporated drug was not sufficient to inhibit the growth of *Issatchenkia orientalis*. In contrast, the complex-loaded fibers (IId) showed a clear and homogenous zone of inhibition (Figure 11F). In the inhibition zone center, the yellow remain lens components were visible. After lens application, the hydrogel was formed and the drug diffusion to the environmental media was reduced caused by the higher viscosity inside the system. In contrast to the AmpB solution, where the diffusion into the agar media was facilitated and larger inhibition zones were obtained, the investigated lenses showed a reduced inhibition zone. These experiments underline that the AmpB concentration of the sodium cholate-loaded fibers (IIb) was not sufficient to overcome microbiotic growth. Instead, the developed polyelectrolyte complex inside the electospun fibers (IId) showed a sufficient antimicrobial effect and was suitable for treatment of keratomycosis.

### Cytotoxicity *In vitro*

**Figure 12:**
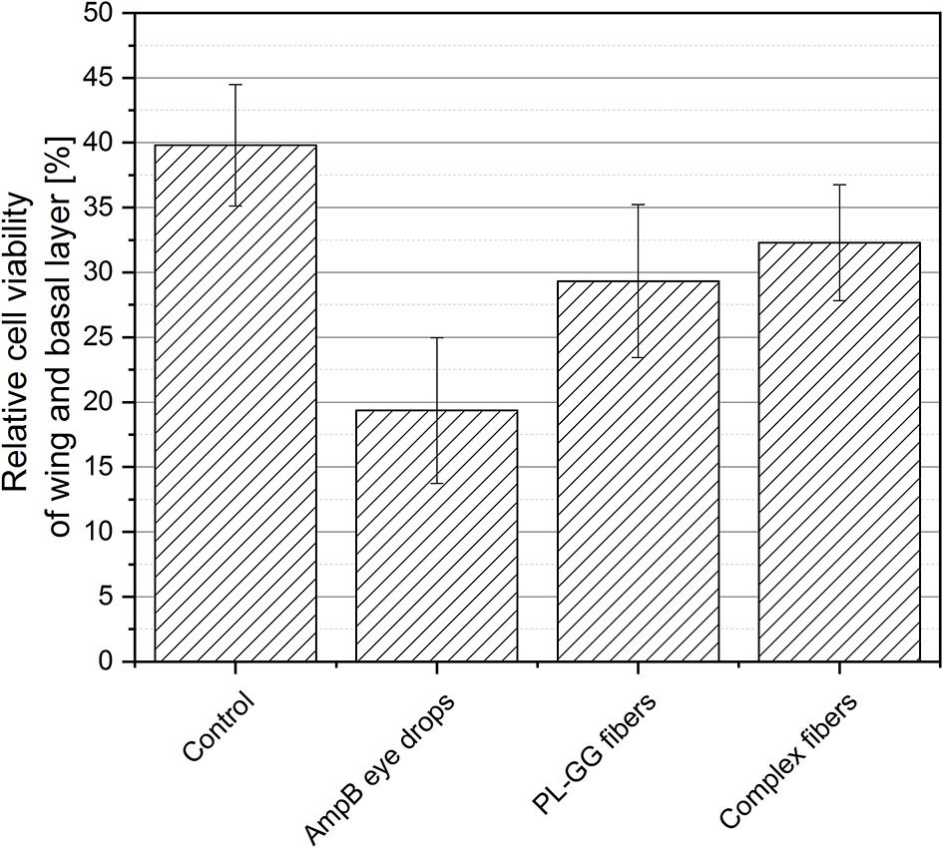
Relative cell viability of wing and basal layers *in vitro*: Untreated cells (control), conventional therapy (AmpB eye drops), 1% Gellan Gum containing Pullulan fibers (PL-GG) and complex loaded Pullulan-Gellan Gum fibers (complex loaded fibers) (median ± SD).

In Figure 12 the results of the cytotoxicity experiments with a multi-stratified epithelium cell model are displayed. The median of different formulations is plotted including the standard derivation. The untreated cells (control) had a viability of 40%. The conventional eye drops had a viability of 19%, the blank Pullulan-Gellan Gum fibers 29% and the complex loaded fibers 32.5%. No difference between the the electrospun samples was obtained. The conventional eye drops instead show a strong decrease of the initial cell viability. The bile salts used for solubilisation of the AmpB in aqueous eye drops as well as the drug itself are characterized by a high cytotoxicity. The developed polyelectrolyte complex is less toxic in the same concentration of free solubilized drug. In the literature similar results were discussed for polyelectrolyte complexes formed with poly(glutamic acid) (Mohamed-Ahmed et al., 2013). These results underline the superiority and higher cell tolerance of the developed polyelectrolyte complex loaded fibers as new innovative antifungal therapy compared with the AmpB eye drops which is currently used in clinical practice.

### Influence of Electron Beam Sterilization

**Figure 13:**
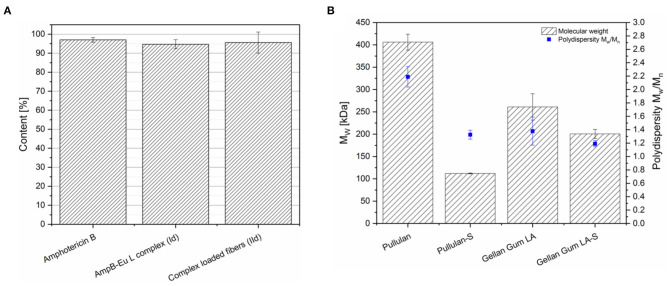
**(A)** Impact of electron beam sterilization onto the Amphotericin B content of different formulations (mean ± SD, *n* = 3). **(B)** Impact of electron beam sterilization on the molecular weight M_w_ and PDI of Pullulan and Gellan Gum before and after sterilization (S) (mean ± SD, *n* = 3).

The pharmacopeia regulates the microbiological status of drug formulations, in which the absence of microbiotics for parenteral and ocular drug delivery systems is especially required. The European Pharmacopeia permits autoclaving, dry heat, aseptic preparation, and sterilization by irradiation. Many polymers, drugs and dosage forms cannot be sterilized by dry or wet heat. Thermal instability and hydrolysis are the major reasons for using the irradiation sterilization. Figure 13A illustrates the effect of the electron beam onto the drug load of pure AmpB, AmpB-Eu L polyelectrolyte complex (Id) and the complex loaded fibers (IId). The AmpB content of pure AmpB decreased to 96.0% of the initial value after sterilization. The drug load of pure polyelectrolyte complex (Id) decreased to 95.0% of the initial value. Complex-loaded fibers (IId) showed a 95.5% AmpB content after sterilization. The complex showed the same stability upon radiation as the pure drug. After sterilization, the residual drug amount is sufficient to treat keratomycosis.

Furthermore, the impact of sterilization on the molecular weight of the polymers was analyzed by AF4 coupled to MALLS (Figure 13B). Pullulan was characterized by 406 kDa molecular weight before sterilization and the molecular weight decreased to 112 kDa, which was ~72% of the initial molecular weight, after sterilization. The polydispersity of Pullulan decreased from 2.19 to 1.33. Instead, Gellan Gum had an initial molecular weight of 261 kDa and 200 kDa after irradiation. The Gellan Gum degradation was with 23% lower in comparison to the spinning copolymer Pullulan. The Gellan Gum polydispersity decreased from 1.38 to 1.19. Both polymers showed a distinct reduction of the molecular weight after electron beam treatment. Also polydispersity decreased, because the polymer chains with higher molecular weight started more fragmentation and as consequence the chain length of the polymer become equal. As result, the polymer size distribution seems to become narrower after sterilization.

## Conclusion

Our research demonstrates different opportunities to load *in situ* gelling nanofibers consisting of Pullulan and Gellan Gum with AmpB prepared from aqueous solution. Therefore, the drug was spun without any further solubilization ingredient (IIa), under addition of sodium cholate as solubilization angent (IIb), drug-loaded PLGA nanoparticles (IIc) and using a polyelectrolyte complex (IId). A reproducible encapsulation of AmpB into PLGA nanoparticles (Ic) with a narrow size distribution followed by incorporation into the fibers was succeeded. Furthermore, we demonstrate the formation of a polyelectrolyte complex (Id) between AmpB and Eu-L, which achieved amorphous distribution of the drug as determined by X-ray diffraction. All electrospun fibers showed mean fiber diameters in the middle nanometer range. However, inhibition of *Issatchenkia orientalis* was insufficient for sodium cholate-solubilized AmpB in the fibers (IIb), whereas the complex-loaded fibers (IId) provided a significant inhibition of fungal growth. The cytoxic experiments *in vitro* underline the good cellular tolerance of the electrospun complex with superiority to the conventional eye drop formulation. Finally, the complex-loaded fibers (IId) showed sufficient resistance against electron beam irradiation thus facilitating terminal sterilization of the drug delivery system.

## Data Availability Statement

The raw data supporting the conclusions of this article will be made available by the authors, without undue reservation.

## Author Contributions

BG designed the study, performed the formulation development and physico-chemical characterization, and wrote the first draft of the manuscript. HL and BG investigated the samples in the plate diffusion experiments. FS captured the ESEM images of the prepared samples. WK treated the samples with the electron beam. JK carried out AF4/MALLS measurements. JH investigated the cytotoxicity *in vitro*. AV and KM finalized the writing of the manuscript and did act as supervisors of the present research. All authors contributed to the article and approved the submitted version.

## Conflict of Interest

The authors declare that this study received no financial funding. The used polymers Eudragit, and Resomer were free samples from Evonik Industries (Darmstadt, Germany) AG. Pullulan was a gift from Nagase GmbH (Düsseldorf, Germany) and Expansorb 5-88 was a gift from Merck KGaA (Darmstadt, Germany). These companies were not involved in the study design, collection, analysis, interpretation of data, the writing of this article or the decision to submit it for publication.

## References

[B1] Angra P. K. Oettinger C. Balakrishna Pai S. D'Souza M. J. (2009). Amphotericin B microspheres: a therapeutic approach to minimize toxicity while maintaining antifungal efficacy. J. Microencapsul. 26, 580–587. 10.3109/02652040902797516 19839793

[B2] Behrens-Baumann W. Finis D. Mackenzie C. Roth M. Geerling G. (2015). Keratomykose - therapiestandards und aktuelle entwicklungen. Klin. Monbl. Augenheilkd. 232, 754–764. 10.1055/s-0035-1546032 26084964

[B3] Bharathi M. J. Ramakrishnan R. Meenakshi R. Padmavathy S. Shivakumar C. Srinivasan M. (2007). Microbial keratitis in south india: influence of risk factors, climate, and geographical variation. Ophthal. Epidemiol. 14, 61–69. 10.1080/09286580601001347 17464852

[B4] Bhartiya P. Daniell M. Constantinou M. Islam F. M. A. Taylor H. R. (2007). Fungal keratitis in Melbourne. Clin. Exp. Ophthalmol. 35, 124–130. 10.1111/j.1442-9071.2006.01405.x 17362452

[B5] Bourcier T. Sauer A. Dory A. Denis J. Sabou M. (2017). Fungal keratitis. J. Fr. Ophtalmol. 40, e307–e313. 10.1016/j.jfo.2017.08.001 28987448

[B6] Bourcier T. Touzeau O. Thomas F. Chaumeil C. Baudrimont M. Borderie V.. (2003). Candida parapsilosis keratitis. Cornea 22, 51–55. 10.1097/00003226-200301000-00012 12502949

[B7] Dangi J. S. Vyas S. P. Dixit V. K. (1998). Effect of various lipid-bile salt mixed micelles on the intestinal absorption of amphotericin-b in rat. Drug Dev. Ind. Pharm. 24, 631–635. 10.3109/03639049809082364 9876507

[B8] Deitzel J. M. Kleinmeyer J. Harris D. Beck Tan N. C. (2001). The effect of processing variables on the morphology of electrospun nanofibers and textiles. Polymer 42, 261–272. 10.1016/S0032-3861(00)00250-0

[B9] Díaz-Valle D. Del Castillo J. M. B. Amor E. Toledano N. Carretero M. M. Díaz-Valle T. (2002). Severe keratomycosis secondary to scedosporium apiospermum. Cornea 21, 516–518. 10.1097/00003226-200207000-00015 12072729

[B10] Espada R. Valdespina S. Alfonso C. Rivas G. Ballesteros M. P. Torrado J. J. (2008). Effect of aggregation state on the toxicity of different amphotericin B preparations. Int. J. Pharm. 361, 64–69. 10.1016/j.ijpharm.2008.05.013 18599228

[B11] Farrell S. McElnea E. Moran S. Knowles S. Murphy C. C. (2017). Fungal keratitis in the Republic of Ireland. Eye 31, 1427–1434. 10.1038/eye.2017.82 28524886 PMC5639195

[B12] Filipe V. Hawe A. Jiskoot W. (2010). Critical evaluation of nanoparticle tracking analysis (NTA) by NanoSight for the measurement of nanoparticles and protein aggregates. Pharm. Res. 27, 796–810. 10.1007/s11095-010-0073-2 20204471 PMC2852530

[B13] Furrer P. Mayer J. M. Plazonnet B. Gurny R. (2002). Ocular tolerance of absorption enhancers in ophthalmic preparations. AAPS PharmSci. 4, 6–10. 10.1208/ps040102 12049486 PMC2751287

[B14] Furrer P. Plazonnet B. Mayer J. M. Gurny R. (2000). Application of *in vivo* confocal microscopy to the objective evaluation of ocular irritation induced by surfactants. Int. J. Pharm. 207, 89–98. 10.1016/S0378-5173(00)00540-8 11036234

[B15] Ghosh S. Das S. De A. K. Kar N. Bera T. (2017). Amphotericin B-loaded mannose modified poly (d, l -lactide-co-glycolide) polymeric nanoparticles for the treatment of visceral leishmaniasis: *in vitro* and *in vivo* approaches. RSC Adv. 7, 29575–29590. 10.1039/C7RA04951J

[B16] Göttel B. de Souza e Silva J. M. Santos de Oliveira C. Syrowatka F. Fiorentzis M. Viestenz A.. (2020). Electrospun nanofibers – A promising solid *in-situ* gelling alternative for ocular drug delivery. Eur. J. Pharm. Biopharm. 146, 125–132. 10.1016/j.ejpb.2019.11.012 31816391

[B17] Green M. Apel A. Stapleton F. (2008). Risk factors and causative organisms in microbial keratitis. Cornea 27, 22–27. 10.1097/ICO.0b013e318156caf2 18245962

[B18] Holden B. A. Sweeney D. F. Sanderson G. (1984). The minimum precorneal oxygen tension to avoid corneal edema. Investig. Ophthalmol. Vis. Sci. 25, 476–480. 6706510

[B19] Kim Y. T. Shin B. K. Garripelli V. K. Kim J. K. Davaa E. Jo S.. (2010). A thermosensitive vaginal gel formulation with HPγCD for the pH-dependent release and solubilization of amphotericin B. Eur. J. Pharm. Sci. 41, 399–406. 10.1016/j.ejps.2010.07.009 20654712

[B20] Lemke A. Kiderlen A. F. Kayser O. (2005). Amphotericin B. Appl. Microbiol. Biotechnol. 68, 151–162. 10.1007/s00253-005-1955-9 15821914

[B21] Mahdy R. A. Nada W. M. Wageh M. M. (2010). Topical amphotericin B and subconjunctival injection of fluconazole (Combination Therapy) versus topical amphotericin B (Monotherapy) in treatment of keratomycosis. J. Ocul. Pharmacol. Ther. 26, 281–285. 10.1089/jop.2010.0005 20565316

[B22] Mohamed-Ahmed A. H. A. Les K. A. Seifert K. Croft S. L. Brocchini S. (2013). Noncovalent complexation of amphotericin-B with poly(α-glutamic acid). Mol. Pharm. 10, 940–950. 10.1021/mp300339p 23234235

[B23] Moustafine R. I. Margulis E. B. Sibgatullina L. F. Kemenova V. A. Mooter G. Van den (2008). Comparative evaluation of interpolyelectrolyte complexes of chitosan with Eudragit® L100 and Eudragit® L100-55 as potential carriers for oral controlled drug delivery. Eur. J. Pharm. Biopharm. 70, 215–225. 10.1016/j.ejpb.2008.04.008 18691856

[B24] Nielsen S. E. Nielsen E. Julian H. O. Lindegaard J. Højgaard K. Ivarsen A.. (2015). Incidence and clinical characteristics of fungal keratitis in a Danish population from 2000 to 2013. Acta Ophthalmol. 93, 54–58. 10.1111/aos.12440 24836583

[B25] Roth M. Daas L. Renner-Wilde A. Cvetkova-Fischer N. Saeger M. Herwig-Carl M.. (2019). The German keratomycosis registry: initial results of a multicenter survey. Ophthalmologe 116, 957–966. 10.1007/s00347-019-0871-9 30810837

[B26] Stefansson E. Foulks G. N. Hamilton R. C. (1987). The effect of corneal contact lenses on the oxygen tension in the anterior chamber of the rabbit eye. Investig. Ophthalmol. Vis. Sci. 28, 1716–1719. 3654146

[B27] Torrado J. J. Espada R. Ballesteros M. P. Torrado-Santiago S. (2008). Amphotericin B formulations and drug targeting. J. Pharm. Sci. 97, 2405–2425. 10.1002/jps.21179 17893903

[B28] Uyar T. Besenbacher F. (2008). Electrospinning of uniform polystyrene fibers: the effect of solvent conductivity. Polymer 49, 5336–5343. 10.1016/j.polymer.2008.09.025

[B29] Van de Ven H. Paulussen C. Feijens P. B. Matheeussen A. Rombaut P. Kayaert P.. (2012). PLGA nanoparticles and nanosuspensions with amphotericin B: potent *in vitro* and *in vivo* alternatives to fungizone and AmBisome. J. Control. Release 161, 795–803. 10.1016/j.jconrel.2012.05.037 22641062

