# Supplementary material for: In situ Gelling Amphotericin B Nanofibers: A New Option for the Treatment of Keratomycosis
Source: Front Bioeng Biotechnol. 2020 Dec 21;8:600384. doi: 10.3389/fbioe.2020.600384 (PMC7786432; doi:10.3389/fbioe.2020.600384)
Supplement: Supplementary file 1 [file Data_Sheet_1.pdf]

Supplementary Material

1 Supplementary Data

1.1 Oscillation Rheology

The rheological measurements of the polymer solutions were carried out by the Kinexus lab+® of Malvern® (Kassel, Germany). 100 mg of complex loaded fibers (0.7 % Amphotericin B), pure Pullulan fibers and Pullulan-Gellan Gum fibers were hydrated with 250 µl STF pH 7.4. All experiments were performed at 34 °C with a 20 mm plate geometry. The frequency sweep was performed over 1-20 Hz with a deformation of 0.4 %. All measurements were performed in the linear viscoelastic region determined previous.

In Supplementary Figure 1A the loss angle of the samples is shown. Pure Pullulan is characterized by a loss angle > 45° indicating properties of viscoelastic fluids. In contrast to this the blank Gellan Gum as well as the complex loaded fibers had a loss angle < 45°, which indicates gel-like behavior. Furthermore, underlines the complex viscosity in B), that the Gellan Gum containing formulations had a higher viscosity in comparison to the fibers without gelling agent. The pure Pullulan fibers had a complex viscosity below 0.2 mPas. The Gellan Gum and complex containing fibers show a shear thinning effect with increasing frequency from 2 mPas to nearly 0.

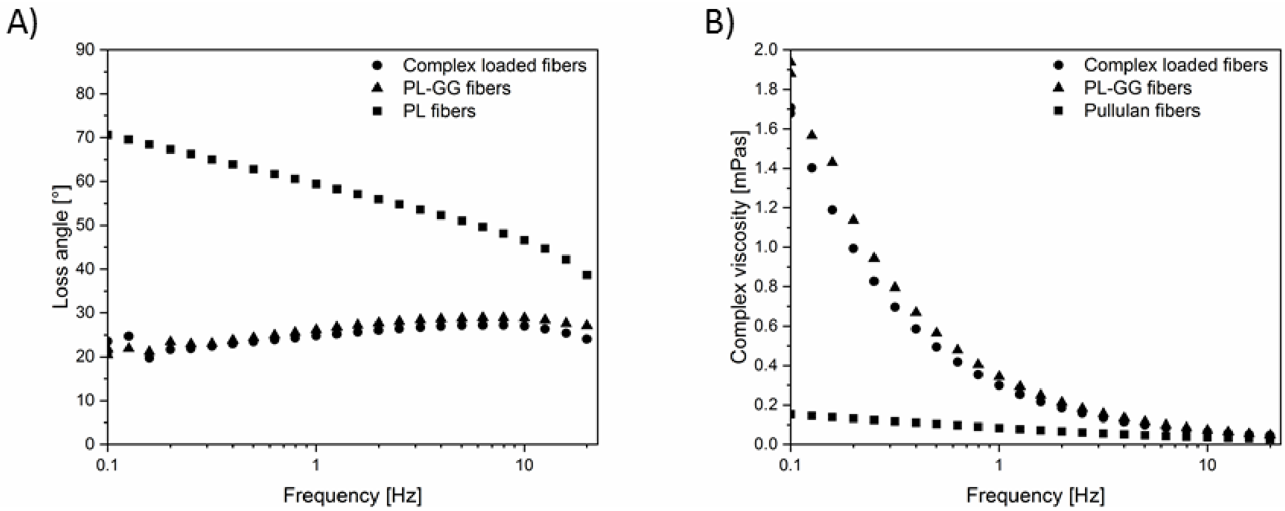

Supplementary Figure 1. Loss angle (A) and complex viscosity dependent from the applied shear frequency of complex loaded fibers, pure Pullulan (PL) and Pullulan-Gellan Gum (PL-GG) fibers.

1.2 Gelling behavior

50 mg of unloaded Pullulan-Gellan Gum fibers and complex loaded fibers (0.7 % Amphotericin B) were hydrated with 500 µl STF pH 7.4 at room temperature in glass vials. After hydration the vials were turned upside down and a picture was taken. In Supplementary Figure 2 the image is displayed.

The image illustrates that both hydrated fibers resist against the gravimetric force, which underline that the addition of polyelectrolyte complex do not have any influence onto the gelation properties.

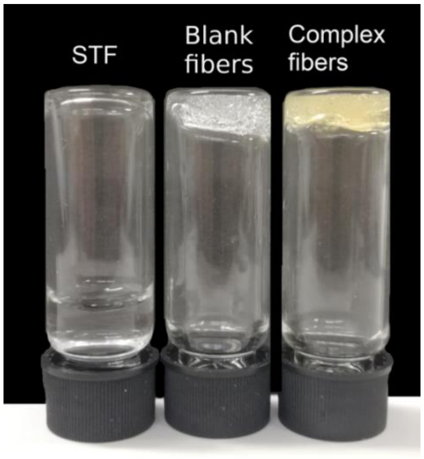

Supplementary Figure 2. Gelling behavior of blank Pullulan-Gellan Gum fibers and complex loaded fibers with STF pH 7.4 at room temperature.

1.3 Transparency

To determine the transparency of the electrospun samples blank Pullulan-Gellan Gum fibers and complex loaded fibers were cut into 1.5 cm lenses with 4 mg weight. The lenses were hydrated on glass dishes with 50 µl STF pH 7.4 at room temperature.

The image is shown in Supplementary Figure 3. Both formulations show that the pattern of the background paper is visible beyond the formed gel. This properties ensure sufficient optical transparency of the gels. The yellow color of the complex loaded fibers result from the incorporated Amphotericin B which is characterized by yellow color of educt.

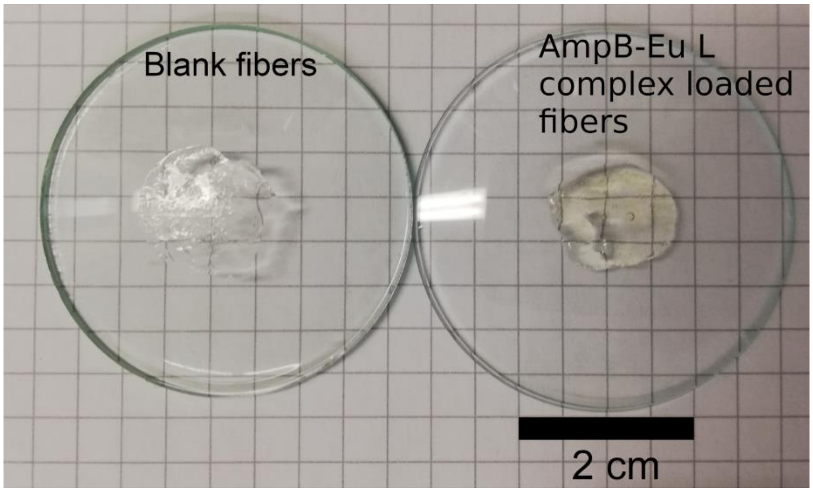

Supplementary Figure 3. Transparency of Pullulan-Gellan Gum (blank) and complex loaded fibers after hydration with STF pH 7.4 at room temperature.
